# Inherent stochasticity, noise and limits of detection in continuous and time-gated fluorescence systems

**DOI:** 10.1371/journal.pone.0313949

**Published:** 2024-12-23

**Authors:** Nicholas H. Vitale, Arjang Hassibi, Hyongsok Tom Soh, Boris Murmann, Thomas H. Lee

**Affiliations:** 1 Department of Electrical Engineering, Stanford University, Stanford, California, United States of America; 2 Department of Radiology, Stanford University, Stanford, California, United States of America; 3 Department of Electrical Engineering, University of Hawaii at Manoa, Honolulu, Hawaii, United States of America; Universiti Brunei Darussalam, BRUNEI DARUSSALAM

## Abstract

We present a model for the noise and inherent stochasticity of fluorescence signals in both continuous wave (CW) and time-gated (TG) conditions. When the fluorophores are subjected to an arbitrary excitation photon flux, we apply the model and compute the evolution of the probability mass function (pmf) for each quantum state comprising a fluorophore’s electronic structure, and hence the dynamics of the resulting emission photon flux. Both the ensemble and stochastic models presented in this work have been verified using Monte Carlo molecular dynamic simulations that utilize the Gillespie algorithm. The implications of the model on the design of biomolecular fluorescence detection systems are explored in three relevant numerical examples. For a given system, the quantum-limited signal-to-noise ratio (QSNR) and limits of detection are computed to demonstrate how key design tradeoffs are quantified. We find that as systems scale down to micro- and nano- dimensions, the interplay between the fluorophore’s photophysical qualities and use of CW or TG has ramifications on optimal design strategies when considering optical component selection, measurement speed, and system energy requirements. While CW systems remain a gold standard, TG systems can be leveraged to overcome cost and system complexity hurdles when paired with the appropriate fluorophore.

## I. Introduction

Fluorescence spectroscopy is a fundamental analysis technique used to identify, quantify, and continually monitor molecular interactions [1] as well as biological micro- and nano-constructs [2]. In the past two decades, the widespread adoption of fluorescence-based techniques in biotechnology has enabled many detection platforms that are ubiquitous today. Notable examples are high-density microarrays [3], quantitative polymerase chain reaction (qPCR) systems [4], and whole genome DNA sequencers [5]. Furthermore, such platforms have found utility beyond pure life science research and in molecular diagnostics to, for instance, detect unique genetic sequences of viruses and bacteria [6] or oncogenic mutations within human cells [7].

Historically, the initial adoption of fluorescence spectroscopy was stifled by two major impediments. The first is related to the practicality of the fluorescence reporters (fluorophores), specifically their restricted commercial availability [8], chemical and photochemical instability [9], and the limited spectra (colors) that they could offer [10]. The second is related to the inherent complexity, bulkiness, and overall cost of the optical instrumentation necessary for fluorescence analysis (i.e., excitation source, filters and lens systems, and photodetectors) [11, 12]. Throughout the years, advancements in photochemistry and molecular engineering have gradually addressed the former. Today, there is an impressive variety of commercially available fluorophores with optimized photophysical qualities that can be readily incorporated in almost any application [13, 14]. Yet, the complexity and cost of the instrumentation is still considered a hinderance and many modern fluorescence spectroscopy systems use relatively expensive laboratory-grade equipment thus limiting the feasibility for mass deployment.

In recent years, the advent of solid-state optoelectronic devices, specifically light emitting diodes (LEDs) [15], laser diodes (LDs) [16], silicon detectors [17], and CMOS scientific cameras [18], has created a unique opportunity to decisively address the instrumentation challenges of fluorescence spectroscopy. While solid-state optoelectronic devices can replace certain bulky optical components (e.g., arc lamps, gas lasers, and cooled photomultiplier tubes) at a fraction of the cost [19], their implementation calls for rigorous designs with system architectures that are compatible with solid-state device manufacturing and development. Such design approaches become increasingly challenging when the detection system is scaled down to realize micro- and nano-sensors. To do this, one must be able to not only model the behavioral dynamics of the fluorescence signals, but also understand the inherent uncertainty and noise to identify optimal designs within the architecture design space.

In this work, we construct a mathematical model to describe the ensemble average and stochastic dynamics of fluorescence through the use of the Markov Chain Model (MCM) and Monte Carlo molecular dynamic simulation techniques. By adopting this systematic approach, we can couple the photophysical qualities of a given fluorophore with the specification of the optical sensors and involved fluorescence instrumentation to identify the limitations as well as achievable detection performances. Specifically, we apply our model to generate closed-form expressions that quantify the quantum-limited signal-to-noise (QSNR) and signal-to-noise (SNR) for both continuous-wave (CW) and time-gated (TG) fluorescence methods. Through numerical examples, we furthermore demonstrate how this framework can be used to identify and establish quantitative tradeoffs in a variety of relevant applications in modern biotechnology.

This work is organized as follows. In Section II, an overview of the quantum mechanical phenomena governing fluorescence is provided. Subsequently in Section III, we introduce a stochastic modeling framework to compute the ensemble average (expected) and stochasticity of fluorescence systems in both CW and TG conditions. These models thoroughly account for a fluorophore’s intra-molecular processes and can be employed in all detection regimes, ranging from systems comprised of many fluorophores in which the quantum noise is essentially “averaged out” to systems with a small number of fluorophores in which “the noise is the signal”. To construct these models, we first describe the system’s expected behavior by establishing a set of ordinary differential equations (ODE) which account for the core quantum mechanical processes illustrated in the Jablonski energy diagram. By analytically solving the ODEs, we derive closed-form approximations to quantify particular characteristics of the system’s expected behavior when subjected to an arbitrary photonic excitation source. These metrics include emission photon flux, quantum state occupancy distribution, system response time-constants, and number of collected photons.

In Section IV, we quantify the randomness of the system and account for the probabilistic nature of fluorescence by building a homogenous continuous-time MCM based on the lifetimes associated with each quantum state. The analytical solutions of the MCM reveal the temporal evolution of the probability mass functions (pmf) that are associated with each quantum state. Subsequently, we compute the uncertainty and noise of the total collected photons for CW and TG measurements. Moreover, we prove that the emission photon flux, unlike other classical photonic sources, is not a Poisson random process. Finally, all the predicted ensemble and stochastic behavior are computationally verified using classical molecular dynamic simulation techniques that rely on the Gillespie algorithm.

Finally, in Section V, we utilize our model in three practical examples to analyze different fluorescence-based molecular detection systems that are subject to unique design constraints such as scaling, imperfect (non-ideal) optics, and stochastic molecular interactions. The underlying objective in each example is to identify the fundamental limits of detection, compute signal-to-noise ratio (SNR) and rationalize design tradeoffs to facilitate the optimal use of design resources and successful extraction of the targeted molecular information.

## II. Quantum processes of fluorescence

**Fig 1:**
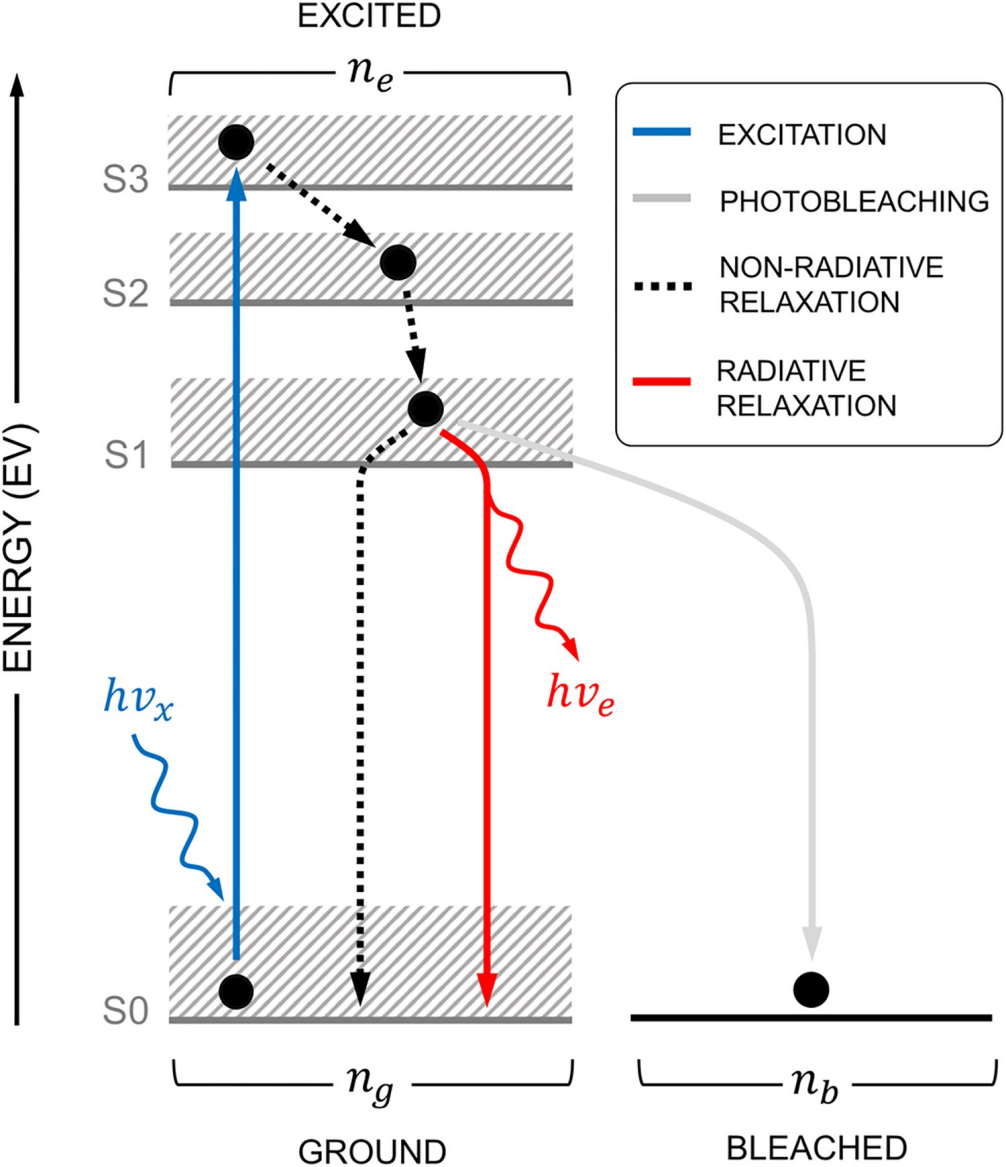
Abbreviated Jablonski energy diagram depicting intra- and extra-molecular quantum mechanical processes involved in fluorescence.

The unique fluorescence phenomena observed in specific molecular structures, generally referred to as a fluorophore, is a product of three quantum mechanical processes which are typically depicted using the Jablonski energy diagram (**Fig 1**) [20]. In the ground state, *n*_*g*_, the electrons constituting the electronic structure of the fluorophore predominately reside in spin-paired orientation at the lowest energy level (*S*_0_). However, when a photon with energy *hυ*_*x*_ couples to and is absorbed by the fluorophore, the excess energy promotes an electron into the excited state, *n*_*e*_, which is comprised of higher energy levels (*S*_1_,*S*_2_ or *S*_3_). This process is referred to as excitation. In the second process, the excited electron descends into lower energy levels and eventually relaxes back to the *S*_0_ state through two mechanistically identical non-radiative relaxation processes, vibrational relaxation (VR) and internal conversion (IC). Both processes are endothermic and exothermic (i.e., absorb and produce heat). Alternatively, in the third process, an excited electron can emit a photon with energy of *hυ*_*e*_ where *hυ*_*x*_ > *hυ*_*e*_ rather than undergoing one of previous non-radiative processes when relaxing from *S*_1_ to *S*_0_. This process is called radiative relaxation, and the combination of all three processes are collectively known as fluorescence. It is important to note that some fluorophores exhibit the presence of triplet states [21] which contribute to the phenomena of phosphorescence, a long-lived version of fluorescence.

The states mentioned above account for intra-molecular quantum processes. However, in practice, there are extra-molecular interactions provoked by the surrounding physical environment [14]. Such interactions, like fluorescence resonance energy transfer (FRET) [22] and collision quenching [23], can create alternative relaxation paths in addition to IC and VR. Alternatively, other interactions may permanently alter the electronic structure of the fluorophore and its photochemical characteristics. A well-known example of this is photobleaching which prohibits photonic emission from the affected fluorophore altogether [24]. Because of the destructive nature, we account for photobleaching by adding a possible path in the Jablonski diagram (**Fig 1**) from *n*_*e*_ to a permanent bleached state, *n*_*b*_. It is imperative to note that accounting for all possible extra-molecular interactions is a daunting task as there are many possibilities which are all environmentally dependent. Yet, it is common to lump them into non-radiating relaxation or deactivating processes provided that their impact can be quantified.

## III. Expected behavior of fluorophores

### A. System of ordinary differential equations

The ensemble average behavior of the fluorescence signal generated by a system comprised of *N* fluorophores can be described by a set of first-order ordinary differential equations (ODEs) [25]. Each ODE describes the change in the number of fluorophores in each specified quantum state (e.g., *n*_*i*_ denotes the number of fluorophores in state *i*) according to the influx and outflux between all other connected states. The general form for each ODE is

$$\frac{dn_{i}}{dt}=\sum_{j,i=1,j\neq i}^{m}\left(r_{j\to i}n_{j}-r_{i\to j}n_{i}\right)$$ (1)

where *m* is the total number of states in the system, and *r*_*j*→*i*_,*r*_*i*→*j*_ are the flux rates from state *j* to state *i* and visa versa. While we use flux rates in (1), we will also refer to their equivalent lifetime (half-life), *τ*, where *τ*_*i*_ = 1/*r*_*i*_.

**Fig 2:**
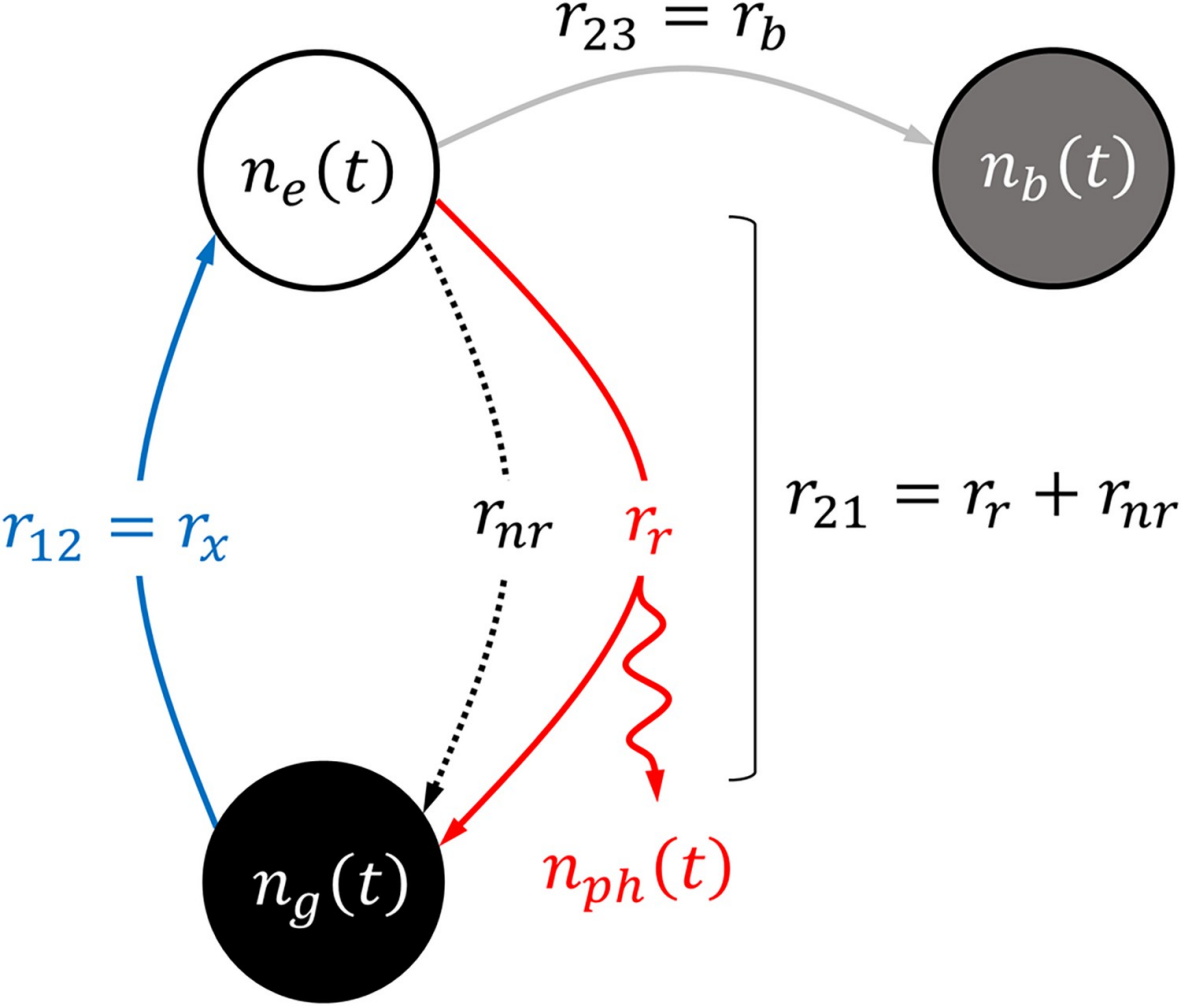
Representative flux path state diagram describing the core quantum states illustrated in the Jablonski energy diagram.

**Fig 2** depicts different flux paths representing the processes illustrated in **Fig 1**. The corresponding system of ODEs describing the rate of change in the number of fluorophores occupying the ground (*n*_*g*_), excited (*n*_*e*_), and bleached (*n*_*b*_) states are listed in **Table 1**, where *n*_*g*_+*n*_*e*_+*n*_*b*_ = *N* at all *t*. As evident, the excitation rate, *r*_*x*_, radiative and non-radiative relaxation rates, *r*_*r*_ and *r*_*nr*_, and the bleaching rate, *r*_*b*_, quantify the rate at which a fluorophore enters and leaves each quantum state. Subsequently, the total number of photons, *n*_*ph*_, emitted over a duration of *T* can be computed from the emission photon flux, *F*_*ph*_, which is a function of the number of fluorophores in the excited state.


$$n_{ph}=\int_{t=0}^{t=T}F_{ph}dt=\frac{r_{r}}{r_{r}+r_{nr}+r_{b}}(r_{r}+r_{nr})\int_{t=0}^{t=T}n_{e}(t)dt$$ (2)

**Table 1:** Expected behavior system of ODEs.

Parameter	Description	Expression
$\frac{dn_{g}}{dt}$	Change in ground state fluorophores	$\left(r_{r}+r_{nr})n_{e}(t)-r_{x}n_{g}(t\right)$
$\frac{dn_{e}}{dt}$	Change in excited state fluorophores	$r_{x}n_{g}(t)-(r_{r}+r_{nr}+r_{b})n_{e}(t)$
$\frac{dn_{b}}{dt}$	Change in bleached state fluorophores	*r*_*b*_*n*_*e*_(*t*)
$\frac{dn_{ph}}{dt}$	Change in emitted photons	*r*_*r*_*n*_*e*_(*t*)

It is important to recognize that *r*_*r*_,*r*_*nr*_, and *r*_*b*_ represent the intra- and extra-molecular relaxation processes and are therefore fluorophore and environment dependent. For most commercially available fluorophores, their values are measured and reported in specific conditions (e.g., in biological buffers and solvents at specific temperatures). In **Table 2**, select fluorophores are listed with commonly reported figure-of-merits (FoM). One common FoM is the fluorescence lifetime, *τ*_*l*_, which describes the average time the fluorophore spends in the excited state before relaxing to ground. Naturally, it is given by the reciprocal of the sum of *r*_*r*_ and *r*_*nr*_. While *r*_*r*_ cannot be measured directly, it can be indirectly assessed from the fluorophore’s quantum efficiency, Φ, which specifies the probability of emitting a photon.


$$\Phi =\frac{r_{r}}{r_{r}+r_{nr}+r_{b}}\approx \frac{r_{r}}{r_{r}+r_{nr}}$$ (3)

**Table 2:** Example fluorophores.

Fluorophore	Solvent^a^	*λ*_*x*_^b^ (nm)	*λ*_*e*_^c^ (nm)	*ϵ*(*λ*_*x*_) (10^3^ M^-1^cm^-1^)	Φ	*τ*_*l*_ (ns)
Fluorescein-isothiocyanate (FITC) ^30^	NaOH	491	510	90.0	0.86	4.11
Ruthenium(II) Complex ([Ru(bpy)_3_]Br_2_) ^31^	CH_3_CN	450	616	12.1	0.063	840
Europium Complex (Eu_3+_L^2^) ^32^	CH_2_Cl_2_	348	483	19.0	0.16	737,000
Enhanced Green Fluorescent Protein (EGFP) ^33^	PBS	489	509	55.0	0.60	2.80

(a) Specified at room temperature (300K), (b) Representative of the wavelength corresponding to the maximum absorbance band, (c) Representative of the wavelength corresponding to the maximum emission band

The rate *r*_*x*_ is somewhat different than other flux rates as it is not a sole function of fluorophore, and it is minimally dependent on the environment. Instead, it is proportional to the incident excitation flux, *F*_*x*_, and the molar extinction coefficient, *ϵ*(*λ*_*x*_), of the fluorophore at wavelength *λ*_*x*_. A simplified formula for *r*_*x*_ is

$$r_{x}=k_{x}F_{x}\epsilon (\lambda_{x})\gamma_{x}$$ (4)

where *γ*_*x*_ is a unit conversion factor and *k*_*x*_ is the proportionality constant that represents the coupling of the electric field of *F*_*x*_ with the dipole within the fluorophore [26]. The *ϵ*(*λ*_*x*_) that is reported in literature describes the ensemble average of fluorophores with uniformly distributed dipole angles [27] in the solution, and therefore we can safely assume *k*_*x*_≈1.

### B. Simulation vs. Closed-form formulations

Numerically simulating the ODEs of **Table 1** can be informative as we can find the dynamic response of the system to any arbitrary *F*_*x*_. More importantly, we can compute *F*_*ph*_ and *n*_*ph*_ for both continuous-wave (CW) or time-gated (TG) measurements. Both CW and TG provide different advantages depending on the application at hand. For example, CW is widely used within biosensor applications whereas TG is more prevalent in fluorescence imaging applications. In CW systems, *n*_*ph*_ is collected by measuring *F*_*ph*_ for *T*_*int*_ seconds under a constant *F*_*x*_. On the other hand, in TG systems, *n*_*ph*_ is collected by measuring *F*_*ph*_ starting at Δ*T*_*D*_ seconds after *F*_*x*_ is turned off, for a duration of *T*_*int*_ seconds. Because *n*_*ph*_ is typically small following one TG measurement, it is common to accumulate *n*_*ph*_ over *K* independent measurements in TG systems.

**Fig 3:**
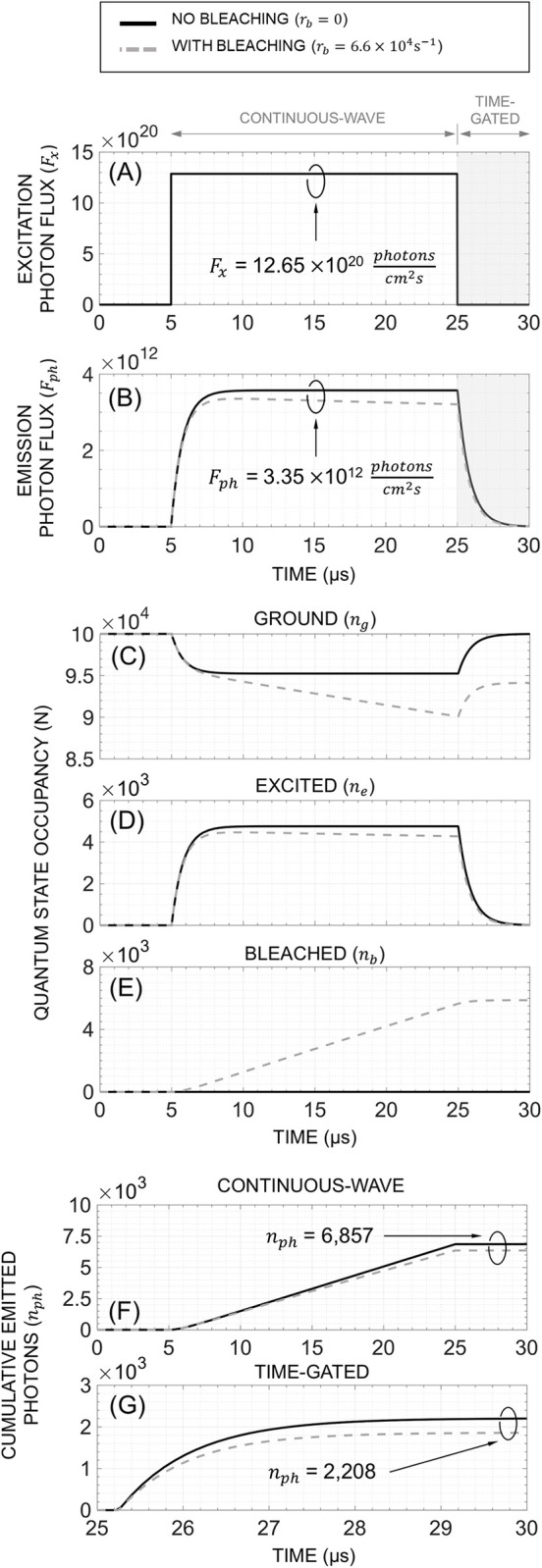
Simulation of the expected behavior for [Ru(bpy)_3_]Br_2_ fluorophores (N = 100,000) subjected to a finite-time excitation pulse in two different bleaching conditions. For CW, T_*int*_ = 20μs, whereas for TG, T_*int*_ = 4.75μs and ΔT_*D*_ = 250ns.

**Fig 3** shows a transient simulation of [Ru(bpy)_3_]Br_2_ fluorophores (*N* = 100,000) that are subjected to a finite-time excitation pulse (see **Fig 3A**). As mentioned previously, fluorophores can experience bleaching which results in irreversible damage. To highlight the impact of this phenomenon, we simulate both CW and TG measurements under two scenarios. In one case, the bleaching is negligible (*r*_*b*_∼ 0), while in the other, bleaching is significant (*r*_*b*_ = 6.66×10^4^ s^-1^). For each bleaching rate, the observed *F*_*ph*_ (**Fig 3B**), quantum state occupancy levels (**Fig 3C** and **3E**), and total number of photons captured (**Fig 3F** and **3G**) are plotted.

As evident, the fluorescence system is inherently non-linear with dynamics that are function of *F*_*x*_. While we can set the rise time of *F*_*x*_ to be extremely fast, the response of the fluorophore in terms of *F*_*ph*_ is slower and dominated by its lifetime with a finite delay before the emission flux reaches steady state. If we consider bleaching to be negligible, then the system finds a steady-state solution resulting in a constant *F*_*ph*_. However, with bleaching there is no steady state *F*_*ph*_ and there is a permanent decline in ground and excited states. In both cases, the *n*_*ph*_ captured under CW linearly increases over the duration of *T*_*int*_ as *F*_*ph*_ is approximately constant during the observation period. On the other hand, the *n*_*ph*_ captured under TG exhibits an exponential increase over the duration of *T*_*int*_ as *F*_*ph*_ follows the exponential relaxation of the excited fluorophores back to the ground state.

In addition to numerically solving the ODEs, we can analytically solve the ODEs in **Table 1** under the assumption of *r*_*b*_∼0. This is a practical approximation in many situations where the excitation power and/or exposure time is low, which is common in many detection platforms. We provide numerical benchmarks for the fluorophores listed **Table 2** and specific closed-form solutions including *n*_*ph*_ in **Table 3**. It is important to note that the formulation for *n*_*ph*_ under CW is in its approximate form which applies for systems where *T*_*int*_ is much greater than the rise-time time constant of *F*_*ph*_.

**Table 3:** Closed-form formulations of ensemble average behavior.

Parameter	Description	Formula	Unit	Fluorophores (N = 100,000)			
				FITC ^e^	[Ru(bpy)_3_]Br_2_^f^	Eu_3+_L_2_^g^	EGFP ^h^
*r*_*x*_	Fluorophore excitation rate ^a,b^	*k*_*x*_*F*_*x*_ϵ(*λ*_*x*_)*γ*_*x*_	$\frac{1}{second}$	1.22×10^7^	5.95×10^4^	67.84	1.76×10^7^
$\bar{n_{g}}$	Number of fluorophores in the ground state	$N\frac{\tau_{l}^{-1}}{r_{x}+\tau_{l}^{-1}}$	NA	95,238			
$\bar{n_{e}}$	Number of fluorophores in the excited state	$N\frac{r_{x}}{r_{x}+\tau_{l}^{-1}}$	NA	4,762			
*F*_*ph*_	Photonic emission flux ^c^	$\Phi \tau_{l}^{-1}\bar{n_{e}}$	$\frac{photons}{second{\;cm}^{2}}$	9.96×10^15^	3.57×10^12^	1.34×10^10^	1.02×10^16^
*τ*_*ph*_	Photonic emission flux rise-time time constant	$\frac{1}{r_{x}+\tau_{l}^{-1}}$	nanoseconds	3.91	800.00	7.02×10^5^	2.66
*n*_*ph*_	Photons collected during continuous-wave operation	*F*_*ph*_(*T*_*int*_−*τ*_*ph*_)	phontons	95,741	6,857	19,123	73,810
	Photons collected during time-gated operation ^d^	${KF}_{ph}\tau_{l}e^{-\frac{{\Delta T}_{D}}{\tau_{l}}}\left(1-e^{-\frac{T_{int}}{\tau_{l}}}\right)$	photons	31,792	2,208	7,010	19,856

(a) *r*_*x*_ is computed given the fluorophores are confined within a surface area of 10^−4^*cm*^2^, (b) γ_x_ = 1000/*A*_*υ*_ where *A*_*V*_ is Avagadro’s number, (c) Computed to represent the isotropic emission given a homogenous fluorophore surface density given by 10^9^/*cm*^2^, (d) Computed for *K* = 10, (e) *F*_*x*_ = 3.53×10^22^ photons/second at *λ*_*x*_ = 491nm, $T_{INT}(CW)=100ns,T_{INT}(TG)=19ns,T_{D}=1ns$, (f) *F*_*x*_ = 1.28×10^21^ photons/second at *λ*_*x*_ *=* 450nm, $T_{INT}(CW)=20\mu s,T_{INT}(TG)=4.75\mu s,T_{D}=250ns$, (g) *F*_*x*_ = 9.43×10^17^ photons/second at *λ*_*x*_ = 348nm, $T_{INT}(CW)=15ms,T_{INT}(TG)=4.75ms,T_{D}=250\mu s$, (h) *F*_*x*_ = 8.48×10^22^ photons/second at *λ*_*x*_ = 489nm, $T_{INT}(CW)=75ns,T_{INT}(TG)=14ns,T_{D}=1ns$

## IV. Stochastic behavior of fluorophores

### A. Deriving Probability Mass Function (PMF)

**Fig 4:**
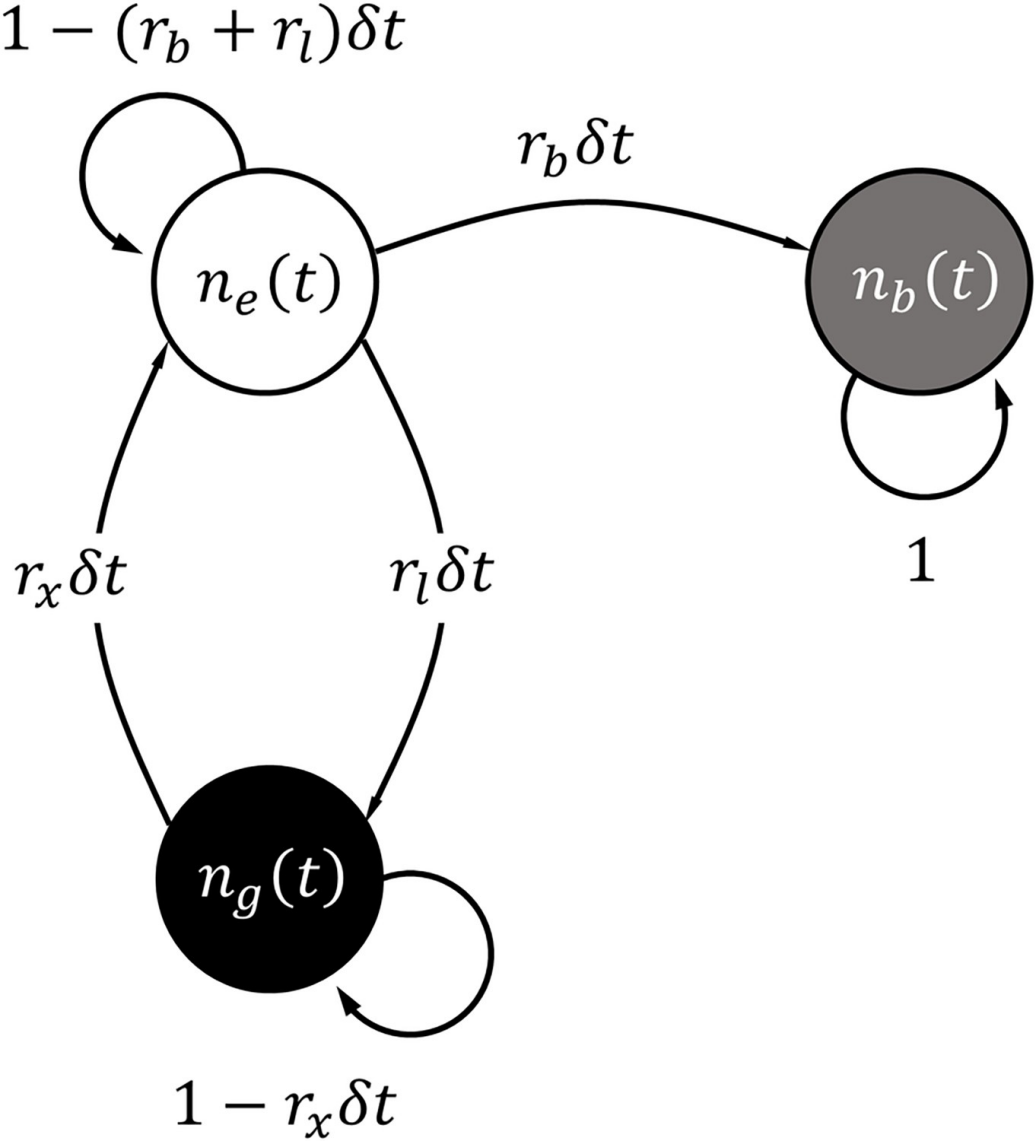
Corresponding Markov Chain Model representing the probability of quantum state transition over an infinitesimal time step.

To quantify the stochasticity of *F*_*ph*_, one needs to consider the lifetimes associated with each quantum state of a fluorophore. Since it is accurate to claim that intra-molecular transitions and most inter-molecular collision events are fundamentally memoryless processes [28], we model the sequence of transitions between quantum states with a homogenous continuous-time Markov Chain Model (MCM) [29]. Each state in the MCM is discrete and one can compute the probability of occupying a state at a time, *t*. Specifically, the probability of occupancy in state *i* is denoted by Pr{*S*(*t*) = *i*}, where $\sum_{k=1}^{m}\mathrm{Pr}\{S(t)=k\}=1$ for a system comprised of *m* states. **Fig 4** depicts the MCM of the example fluorophore system described in **Fig 2,** noting *m* = 3.

To ultimately compute the probability of being in a quantum state at any *t*, we first define the transition probability from state *i* to *j*,*p*_*i*→*j*_, in an infinitesimal period of time, δt, by

$$p_{i\to j}=\mathrm{Pr}\left\{S(t+\delta t)=j|S(t)=i\right\}$$ (5)

which has the following relationship with the flux rate *r*_*i*→*j*_

$$p_{i\to j}=r_{i\to j}\delta t.$$ (6)

By expanding (6) for all states, we create the transition probability matrix, ***P***_*m*×*m*_, of the fluorophore system as

$$P=\left[\begin{array}{cccc} p_{1\to 1} & p_{2\to 1} & \cdots & p_{m\to 1}\\ p_{2\to 1} & p_{2\to 2} & \cdots & \cdots \\ \cdots & \cdots & \ddots & \cdots \\ p_{1\to m} & p_{2\to m} & \cdots & p_{m\to m} \end{array}\right]=\left[\begin{array}{cccc} 1-\lambda_{1}\delta t & r_{2\to 1}\delta t & \cdots & r_{m\to 1}\delta t\\ r_{1\to 1}\delta t & 1-\lambda_{2}\delta t & \cdots & \cdots \\ \cdots & \cdots & \ddots & \cdots \\ r_{1\to m}\delta t & r_{2\to m}\delta t & \cdots & 1-\lambda_{m}\delta t \end{array}\right]$$ (7)

where *λ*_*i*_ is the total outflux rate for state *i*, described by $\lambda_{i}=\sum_{j=1,j\neq i}^{m}r_{i\to j}$. Now, if vector ***S***_*m*×1_ describes the probability mass function (pmf) of states, i.e., its entry *j* is Pr{*S*(*t*) = *j*}, then the temporal evolution of ***S***_*m*×1_ for every δ*t* is

S(t+δt)=PS(t)=(I+Qδt)S(t) (8)

in which ***I*** is an identity matrix, whereas ***Q***, conventionally referred to as the generator matrix, is

$$Q=\left[\begin{array}{cccc} -\lambda_{1} & r_{2\to 1} & \cdots & r_{m\to 1}\\ r_{1\to 1} & -\lambda_{1} & \cdots & \cdots \\ \cdots & \cdots & \ddots & \cdots \\ r_{1\to m} & r_{2\to m} & \cdots & -\lambda_{m} \end{array}\right]$$ (9)

Subsequently, (8) can be rearranged to

$$\frac{S(t+\delta t)-S(t)}{\delta t}=QS(t)$$ (10)

By taking the limit of δ*t*→0, (10) converges to the differential equation that describes the dynamics of ***S*** (referred to as the forward Chapman-Kolmogorov Eq [29])

$$\dot{S}(t)=QS(t)$$ (11)

There are three significances for (11) that are important within the context of this work. The first is that if the probability mass function (pmf) for the states at *t* = 0 is ***S***(0), then ***S***(*t*) at any *t*≥0 can be computed by

$$S(t)=e^{Qt}S(0)$$ (12)

The second is that, if that for any *N* number of fluorophores with pmf of ***S***_*N*_(*t*), we can have ***S***_*N*_(*t*) = *N****S***(*t*). This relationship assumes that fluorophores are statistically independent which is valid for most practical applications in which the concentration of fluorophores is small, i.e., fluorophore-fluorophore interactions are rare. Third, all the parameters required to assemble ***Q*** can be derived by the macroscopic flux path rates depicted in **Fig 2**. Specifically, the representative ***Q*** matrix of system in **Fig 2**, is

$$Q=\left[\begin{array}{ccc} -r_{x} & r_{x} & 0\\ r_{l} & -(r_{l}+r_{b}) & r_{b}\\ 0 & 0 & 0 \end{array}\right]$$ (13)

It is equally critical to note that the PMF given by ***S***(*t*) simultaneously allows one to compute the uncertainty and noise of *F*_*ph*_ and subsequently *n*_*ph*_. However, the validity of ***S***(*t*) given by (12) is based on ***Q*** remaining constant to obey the assumption of homogeneity in the MCM [30]. Specifically, any system parameter that forces ***Q*** to be a function of time, like a change in *F*_*x*_, means that the solution to ***S***(*t*) may not be given exactly by (12). Consequently, the resulting uncertainty and noise of the system must be analyzed separately for CW and TG measurements as *F*_*x*_ inherently changes with time.

### B. Emission fluctuations in CW

Let’s first consider a system in which we want measure *F*_*ph*_ subject to a constant *F*_*x*_, assuming steady state conditions, and negligible bleaching. Here, our main objective is to compute the inherent fluctuations of *F*_*ph*_ to estimate the variation (and uncertainty) in *n*_*ph*_ for a given integration time, *T*_*int*_.

Classically, photons emitted by ideal light sources are known to follow a Poisson random process with emission inter-arrival times that are characterized by an exponential probability density function (pdf) [31]. Nevertheless, this assumption does not apply to a fluorescence system that follows the MCM given in **Section IV. A**. Therefore, to formulate *n*_*ph*_, we first need to formulate the pdf of the photon emission inter-arrival time generated by a single fluorophore.

According to the MCM, the total time it takes for a fluorophore to be excited and subsequently return to its ground state, regardless of photon emission, is the sum of two sequential independent events (i.e., random variables), specifically excitation and relaxation. We represent this total time by the random variable *t*_*θ*_ such that

$$t_{\theta }=t_{x}+t_{l}$$ (14)

where *t*_*x*_ and *t*_*l*_ are exponentially distributed random variables and represent the sojourn times spent in the ground and excited states, respectively. Since *t*_*x*_ and *t*_*l*_ are statistically independent, we can define the pdf of *t*_*θ*_ as a hypoexponential distribution [32], *φ*_*θ*_(*t*), given by

$$\varphi_{\theta }(t)=\frac{r_{x}r_{l}}{r_{l}-r_{x}}(e^{-r_{x}t}-e^{-r_{l}t}).$$ (15)

It is important to recognize that while *t*_*θ*_ represents the inter-arrival time between excitation-relaxation events, it does not account for photon emission. When an excited fluorophore relaxes to its ground state, it has a probability *p* of emitting a photon equal to the quantum efficiency, Φ, which was dervied earlier in (3) as the ratio between *r*_*r*_ and *r*_*l*_. Now by using (3) and (15), we can formulate *φ*_*ph*_(*t*), the pdf of the inter-arrival time between photon emissions by

$$\varphi_{ph}(t)=\sum_{m=1}^{\infty }p{\left(1-p\right)}^{m-1}\psi_{m}$$ (16)

where *ψ*_*m*_ is

$$\psi_{m}=\underset{m}{\underset{︸}{\varphi_{\theta }(t)*\varphi_{\theta }(t)*\varphi_{\theta }(t)*\cdots *\varphi_{\theta }(t)}}$$ (17)

It can be shown that *φ*_*ph*_(*t*) can be simplified into another hypoexponential pdf in the form of

$$\varphi_{ph}(t)=\frac{\lambda_{1}\lambda_{2}}{\lambda_{2}-\lambda_{1}}(e^{-\lambda_{1}t}-e^{-\lambda_{2}t})$$ (18)

where *λ*_1_,*λ*_2_ = 1/2(*r*_*x*_+*r*_*l*_±*γ*) and $\gamma =\sqrt{{\left(r_{x}+r_{l}\right)}^{2}-4\Phi r_{x}r_{l}}$.

By applying (18), we can calculate the mean and variance of *F*_*ph*_ and subsequently the variations of the observed *n*_*ph*_. If we assume that *T*_*int*_ is much larger than the mean inter-arrival time, 〈*φ*_*ph*_(*t*)〉, we can apply the elementary renewal theorem [33] to calculate 〈*F*_*ph*_〉, the average emission photon flux

$$\langle F_{ph}\rangle =\frac{\lambda_{1}\lambda_{2}}{\lambda_{1}+\lambda_{2}}$$ (19)

and $\sigma_{F_{ph}}^{2}$, the variance of the emission photon flux

$$\sigma_{F_{ph}}^{2}=\frac{\lambda_{1}\lambda_{2}(\lambda_{1}^{2}+\lambda_{2}^{2})}{{\left(\lambda_{1}+\lambda_{2}\right)}^{3}}$$ (20)

Consequently, 〈*n*_*ph*_〉, the average of *n*_*ph*_, becomes

$$\langle n_{ph}\rangle =\langle F_{ph}\rangle T_{int}$$ (21)

and $\sigma_{n_{ph}}^{2}$, the variance of *n*_*ph*_

$$\sigma_{n_{ph}}^{2}=\sigma_{F_{ph}}^{2}T_{int}$$ (22)

The formulations of (21) and (22) demonstrate that the ratio between the excitation and relaxation rate, as well as the fluorophore quantum efficiency, strongly influences the observed statistics. For example, if one rate were to dominate relative to the other, *φ*_*ph*_(*t*) would converge to an exponential distribution and result in statistics that follow a Poisson process, i.e., $\langle n_{ph}\rangle =\sigma_{n_{ph}}^{2}$. Yet in most practical systems, fluorophores are excited sufficiently enough that a distinguishable *F*_*ph*_ is ellicited. As a result, the excitation and relaxation rates tend to compete, and the observed statistics generally adhere to $\langle n_{ph}\rangle >\sigma_{n_{ph}}^{2}$. More importantly, this statistical relationship demonstrates that the fluorescence phenomenon follows a sub-Poisson random process.

It is also logical to evaluate the power spectral density (PSD) of *F*_*ph*_ to better understand the spectral characteristics of the fluctuations. To do this, we first model *F*_*ph*_ by *x*(*t*), a random pulse train with *t*_*n*_ denoting the arrival time of its *n*^th^ pulse. The Fourier transform, *X*(*jω*), of this pulse train becomes

$$X(j\omega )=G(j\omega )\sum_{n=1}^{N}e^{-j\omega t_{n}}$$ (23)

where *G*(*jω*) is the Fourier transform of the pulse shape. For the stochastic signal *x*(*t*), the corresponding single-sided PSD is

$$S(\omega )=\underset{T\to \infty }{lim}\frac{2}{T}\langle {\left|X(j\omega )\right|}^{2}\rangle =\underset{T\to \infty }{\lim}\frac{2}{T}{\left|G(i\omega )\right|}^{2}\langle \sum_{m=1}^{N}\sum_{n=1}^{N}e^{-j\omega (t_{n}-t_{m})}\rangle$$ (24)

To take the expectation of the double summation, we introduce the characteristic function, ϕ(*jω*), which is the Fourier transform of *φ*_*ph*_(*t*)

$$\phi (j\omega )=\langle e^{-j\omega t}\rangle =\frac{\lambda_{1}\lambda_{2}}{\left(\lambda_{1}+j\omega )(\lambda_{2}+j\omega \right)}$$ (25)

By substituting (25) into (24), it can be shown that the closed-form single-sided PSD of *x*(*t*) is given by

$$S(\omega )=2\pi \nu^{2}\delta (\omega )+2\nu \langle {\left|G(j\omega )\right|}^{2}\rangle \left(1+2R[\frac{\phi (j\omega )}{1-\phi (j\omega )}]\right)$$ (26)

where *v* is the mean pulse rate of *x*(*t*). If we assume that the pulse shape of *x*(*t*) follows a Dirac-delta function, we can explicitly write (26) in terms of *F*_*ph*_ as

$$S(\omega )=2\pi F_{ph}^{2}\delta (\omega )+2\sigma_{F_{ph}}^{2}\left(\frac{1+\frac{\omega^{2}}{\lambda_{1}^{2}+\lambda_{2}^{2}}}{1+\frac{\omega^{2}}{{\left(\lambda_{1}+\lambda_{2}\right)}^{2}}}\right)$$ (26)

Again, assuming statistical independence of each fluorophore, the corresponding PSD of a system comprised of *N* fluorophores is given by *NS*(*ω*).

**Fig 5:**
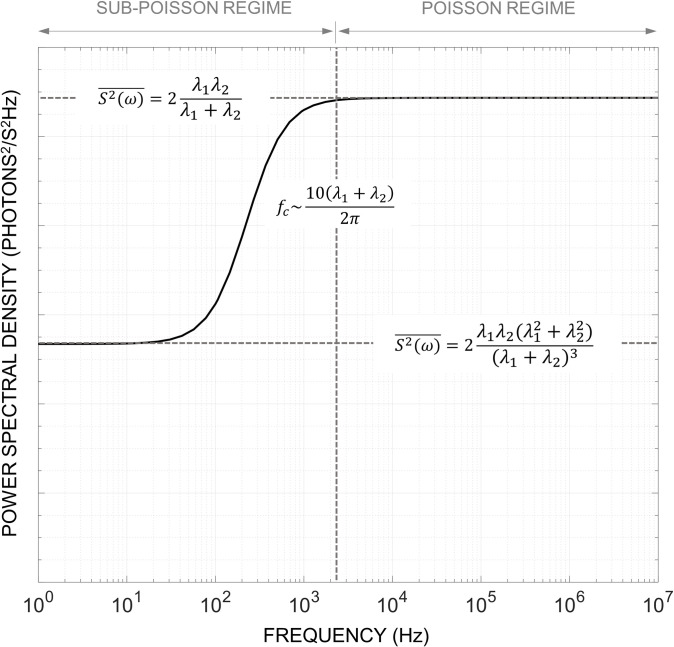
Plot of the predicted power spectral density of fluorescence for an arbitrary fluorophore. The spectrum is divided into its sub-Poisson and Poisson regions by the transition frequency located at f_c_.

**Fig 5** depicts the PSD spectrum for an arbitrary fluorophore. The shape of the resulting PSD (ignoring the DC component), exhibits two distinct regimes which are dependent on the ratio between *r*_*x*_ and *r*_*l*_ as mentioned previously. At low frequencies, *ω*→0, the spectrum follows a sub-Poisson process with a spectral density, i.e., magnitude, equal to $2\sigma_{F_{ph}}^{2}$. However, as the frequency increases, the spectral density begins to rise and eventually converges to 2〈*F*_*ph*_〉 which is characteristic of a Poisson process. This transition occurs around 10 times the frequency location obtained from the numerator of (26) which is given by *f* = (*λ*_1_+*λ*_2_)/2*π*. Since *T*_*int*_ maps to the frequency *f* = 1/2*T*_*int*_, the high frequency behavior of the PSD demonstrates that for a small enough *T*_*int*_ the probability of seeing multiple photons, and thus any correlation between inter-arrival times, diminishes and results in random process that appears Poisson. However, practical applications operate with a *T*_*int*_ that resides in the sub-Poisson regime.

### C. Emission fluctuations in TG

Now, let’s consider a TG system with negligible bleaching where we aim to measure *F*_*ph*_ approximately Δ*T*_*D*_ seconds after *F*_*x*_ is shutoff over the duration *T*_*int*_. Our objective is to predict the variance of the total *n*_*ph*_ resulting from the accumulation of *K* independent TG measurements. Here we make the critical assumption that the on and off duration of *F*_*x*_ is such that in each phase, the distribution of fluorophores in the ground and excited states reach their steady state values before a measurement is made.

To formulate the variance of *n*_*ph*_, we first start by computing the probability that a photon is emitted in the time window [Δ*T*_*D*_,*T*_*int*_] given that there is exactly one excited fluorophore immediately after *F*_*x*_ turns off. Subsequently, we can apply the MCM where ***Q*** is modified with *r*_*x*_,*r*_*b*_ = 0 and ***S***(0) is initialized with one fluorophore in the excited state. It can be shown that the probability of photon emission, *p*, is the integral of the excited state’s pmf given by ***S***(*t*) over the time [Δ*T*_*D*_,*T*_*int*_+Δ*T*_*D*_]

$$p=\Phi \left(e^{-\frac{\Delta T_{D}}{\tau_{l}}}-e^{-\frac{T_{INT}+\Delta T_{D}}{\tau_{l}}}\right)$$ (27)

Now, while (27) represents the probability that a photon is emitted given there is one excited fluorophore, we must account for the fact that every time a measurement is made it is not guaranteed an excited fluorophore is present. As a result, the variance of *n*_*ph*_ is a product of both the uncertainty in *n*_*e*_ and the probability of photon emission.

Assuming that the system is in steady state before *F*_*x*_ shuts off, we can leverage the MCM to formulate 〈*n*_*e*_〉, the probability of there being an excited fluorophore

$$\langle n_{e}\rangle =\frac{r_{x}}{r_{x}+r_{l}}$$ (28)

and $\sigma_{n_{e}}^{2}$, the variance of *n*_*e*_

$$\sigma_{n_{e}}^{2}=\frac{r_{x}}{r_{x}+r_{l}}\left(1-\frac{r_{x}}{r_{x}+r_{l}}\right)$$ (29)

By utilizing (27)–(29), it can be shown through the Burgess Variance Theorem [34] that the mean and variance of *n*_*ph*_ collected over *K* measurements is given by

$$\langle n_{ph}\rangle =Kp\langle n_{e}\rangle$$ (30)

$$\sigma_{n_{ph}}^{2}=K(p^{2}\sigma_{n_{e}}^{2}+\langle n_{e}\rangle p(1-p))$$ (31)

where, again, the statistics of *n*_*ph*_ for a system comprised of *N* fluorophores are given by *N*〈*n*_*ph*_〉 and ${N\sigma }_{n_{ph}}^{2}$.

### D. Molecular dynamic simulation of fluorescence systems

To verify the formulations predicting the ensemble average and stochastic behavior of a fluorescence system, we conduct Monte Carlo molecular dynamic simulations by employing the Gillespie Algorithm (GA). The GA is widely used to simulate exact stochastic trajectories of chemical and biochemical reactions and their participating reactants across a large range of concentrations. In a system where there are *i* different reaction types, the GA determines what reaction, *c*_*i*_, occurred at time *t* and then generates the time to the next reaction, *t*+*τ*, by sampling the probability distribution formed by each reaction’s propensity function, *a*_*i*_(*x*) [35]. Depending on the type of reaction that occurred, the GA tracks the state of each reactant through a corresponding state update vector, *u*_*i*_.

**Fig 6:**
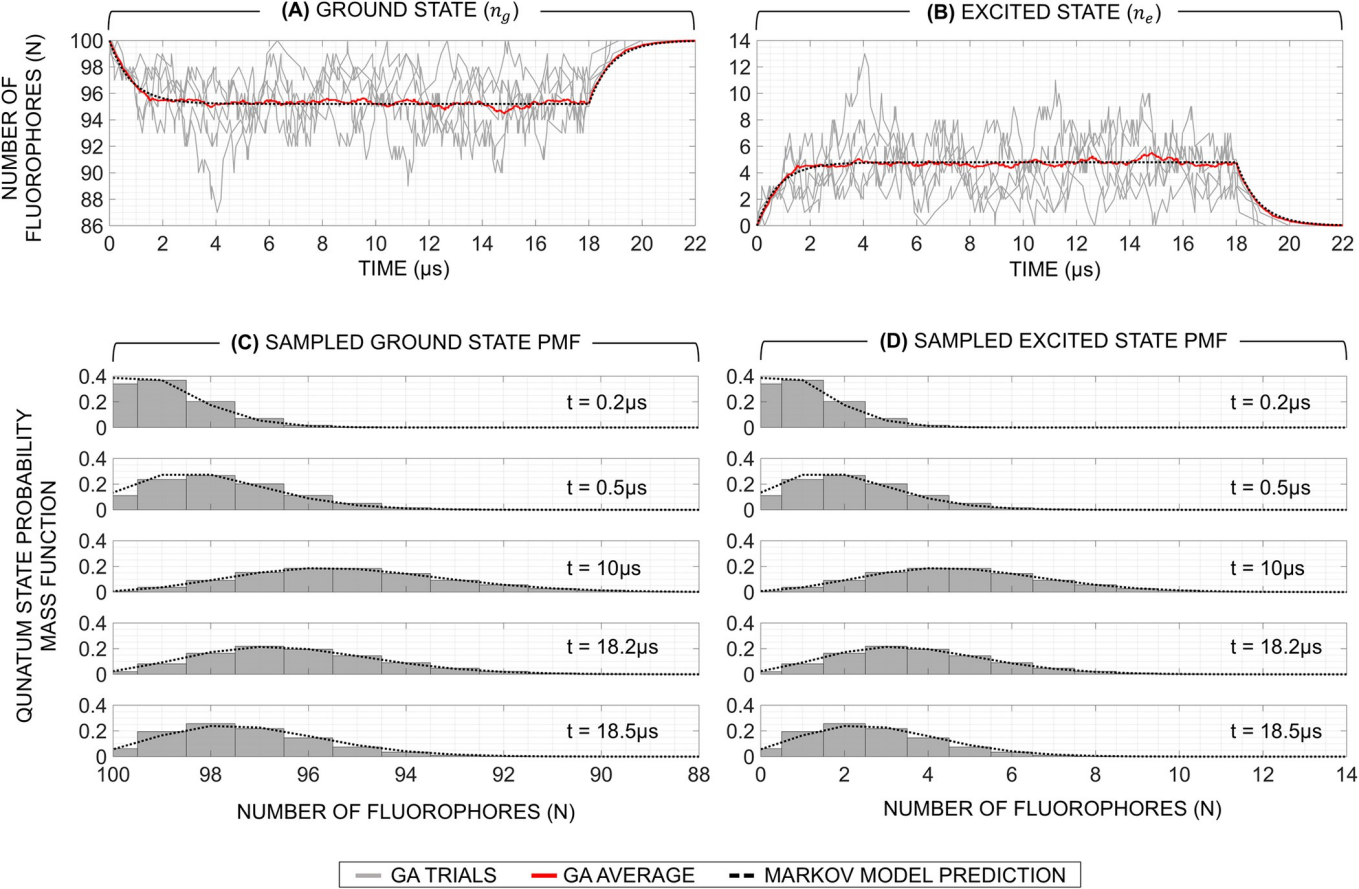
Results of the quantum state occupancy in the exited (A) and ground (B) states and evolution of its pmf over time (C, D) simulated by the Gillespie algorithm (subset shown is 5 trials) compared to that predicted by the Markov model for [Ru(bpy)_3_]Br_2_ fluorophores (N = 100) subjected to the same excitation photon flux as in Fig 3.

**Fig 7:**
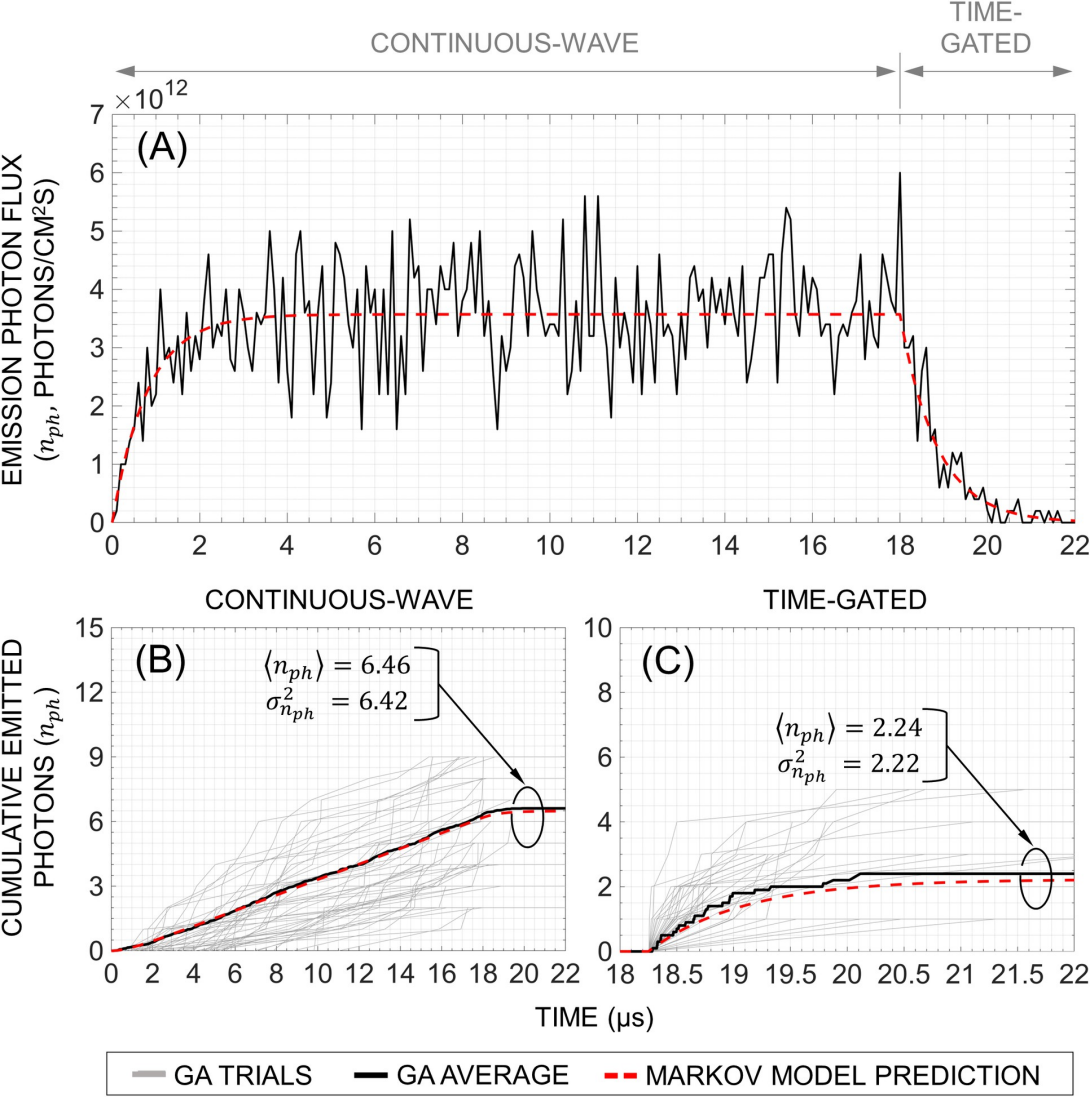
Results of the emission photon flux (A) and cumulative emitted photons (B, C) simulated by the Gillespie algorithm (subset shown is 10 trials) compared to that predicted by the Markov model for [Ru(bpy)_3_]Br_2_ fluorophores (N = 100) subjected to the same excitation photon flux as in Fig 3. For CW, T_*int*_ = 18μs, whereas for TG, T_*int*_ = 3.75 μs and ΔT_*D*_ = 250ns.

In **Table 4,** we associate each reaction, propensity function, and corresponding state update vector with the individual macroscopic flux paths and quantum states of the fluorescence system depicted in **Fig 2.** Based on the simulation parameters of **Table 4**, **Figs 6** and **7** depict the result of 10,000 GA simulation trials of [Ru(bpy)_3_]Br_2_ fluorophores (*N* = 100) subjected to the same excitation photon flux intensity illustrated in **Fig 3** for both CW and TG measurements in the scenario where bleaching is not considered.

**Table 4:** Gillespie algorithm simulation parameters.

Reaction Type (*c*_*i*_)	Propensity Function (*a*_*i*_(*x*))	State-Change Vector (*u*_*i*_)			
		Δ*n*_*g*_	Δ*n*_*e*_	Δ*n*_*b*_	Δ*n*_*ph*_
Excitation	*a*_1_(***x***) = *r*_*x*_*n*_*g*_(*t*)	−1	+1	0	0
Non-Radiating Relaxation	*a*_2_(***x***) = *r*_*nr*_*n*_*e*_(*t*)	+1	−1	0	0
Radiating Relaxation	*a*_3_(***x***) = *r*_*r*_*n*_*e*_(*t*)	+1	−1	0	+1
Bleaching	*a*_4_(***x***) = *r*_*b*_*n*_*e*_(*t*)	0	−1	+1	0

In **Fig 6**, we depict the stochastic trajectories of the ground (**Fig 6A**) and excited (**Fig 6B**) states generated by the GA. The average of the GA trials and the trajectory predicted by the MCM are overlaid to demonstrate the validity of the MCM. Initially, in the first microsecond, the fluctuation of *n*_*e*_ is relatively small due to the fluorophore’s lifetime being on the order of this observation period. For fluorophores with small *τ*_*l*_, the observed fluctuation would increase more rapidly as their response time to an excitation signal is enhanced. After a sufficient number of time-constants have elapsed (~5*τ*_*l*_), the system reaches its steady state. At that point, the fluctuation reaches its maximal value as the average number fluorophores residing in *n*_*e*_ at any given time is approximately constant. In steady state, the fluctuation of *n*_*e*_ scales directly with the intensity of *F*_*x*_ as pointed out in **Section IV.B**. Once the excitation pulse is turned off, the fluctuation of *n*_*e*_ begins to slowly taper as the number of fluorophores decreases at an exponential rate defined by *τ*_*l*_.

In **Fig 6C** and **6D**, histograms of the number of fluorophores in the excited and ground states are plotted at five selected time points during the rising and falling edges of the excitation pulse as well as steady state. These histograms represent the simulated pmf of the quantum states. We overlay the pmf predicted by the MCM and it can be observed that all the simulated pmfs follows the distribution predicted by the MCM at each time point. This further validates that the MCM correctly predicts the temporal evolution of the pmf for a fluorescence system and demonstrates that the GA is a suitable tool for simulating fluorophore populations.

In addition to tracking the quantum state occupancies, *F*_*ph*_ and the subsequent statistics of *n*_*ph*_ for CW and TG can be extracted from the photon emission timing information generated by the GA simulation. In **Fig 7A**, the simulated emission photon flux agrees well with the trajectory predicted by the MCM. On the other hand, **Fig 7B and 7C** depict the simulated trajectories of *n*_*ph*_ under CW and TG conditions, respectively. As expected, CW generates more *n*_*ph*_ on average due to the steady *F*_*ph*_ and longer *T*_*int*_. Alternatively, TG generates less *n*_*ph*_ due to the measurement window being placed during the exponential decay of *F*_*ph*_. It should be noted that the fluctuation observed in *F*_*ph*_ may further increase if subjected to a “noisy” *F*_*x*_, which is not considered in this simulation.

**Fig 8:**
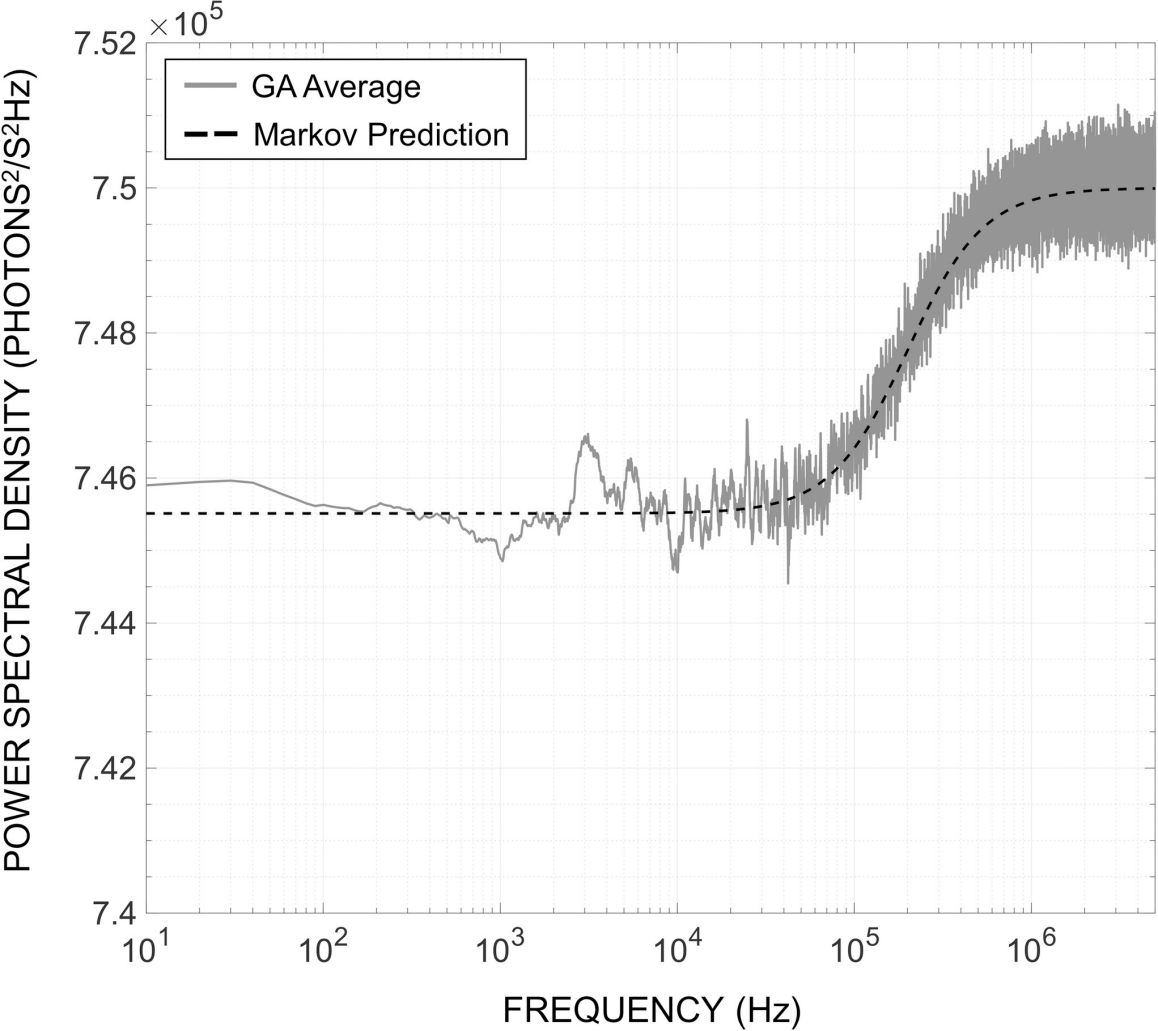
Results of power spectral density simulated by the Gillespie algorithm compared to that predicted by the Markov model for [Ru(bpy)_3_]Br_2_ fluorophores (N = 100) subjected to the same excitation photon flux as in Fig 3.

Finally, we further confirm the validity of the proposed MCM and GA method by constructing the PSD via the simulated photon emission times. **Fig 8,** shows the PSD generated by the GA and that derived in **Section IV.B**. As predicted, the simulated PSD exhibits the transition from sub-Poisson to Poisson behavior around the center frequency determined by the values of *F*_*x*_,*τ*_*l*_, and Φ. More importantly, the observation of the predicted PSD via stochastic numerical simulation validates the hand-derivation of the PSD presented in (26). The agreement between the simulated results of **Figs 6**–**8**, and the predicted expected and stochastic behavior computationally validates the proposed modeling framework for fluorescence systems.

## V. Application to fluorescence system design

### A. Scaling impacts on SNR and measurement speed

In many fluorescence systems, such as DNA microarray assays [36] and next-generation sequencing (NGS), hundreds of thousands to millions of fluorescently labeled spots are imaged on a surface. These systems, which commonly employ CW, encounter a hard tradeoff between the speed at which surfaces can be imaged and signal-to-noise ratio (SNR) that can be achieved. Here, the SNR is defined as the ratio of signal intensity to measured background noise intensity. Many systems aim to achieve minimum SNRs of > 6dB while the maximum SNR is set by either the well-capacity of the imager (typically > 40dB), or maximum image acquisition time [37, 38].

**Fig 9:**
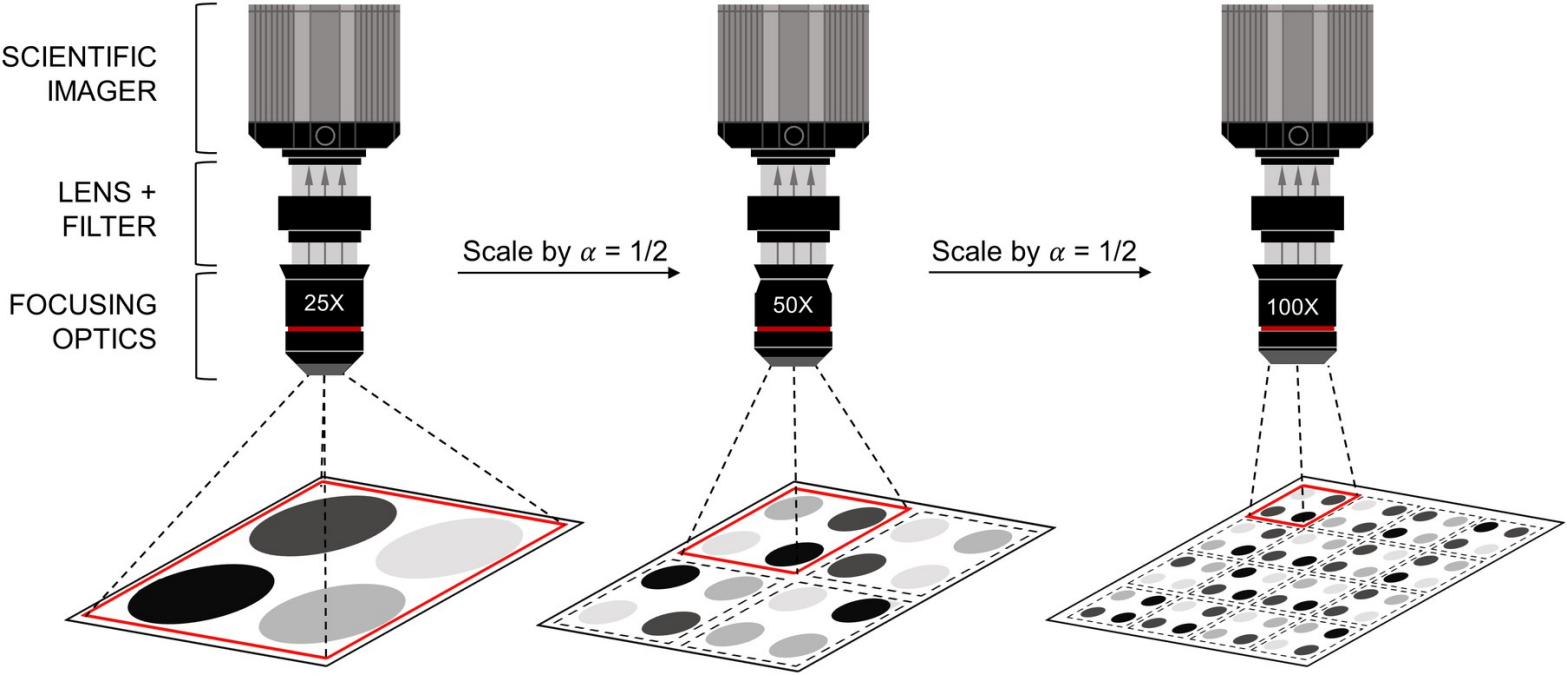
Pictorial representation of isomorphically scaling down spots within a fixed field of view and corresponding imaging configuration.

In recent years, the average size of a spot has isomorphically scaled to achieve higher sample density [39]. However, to image increasingly smaller spots, the accompanying imaging hardware must compensate to maintain a specified target SNR. This is often manifested by adjusting the imaging optics to ensure that each spot sample maps to a certain *M*×*M* region of pixels on the imager (see **Fig 9**). Consequently, this optical adjustment constrains the field of view and can result in an overall longer time to image a surface of a given size.

**Fig 10:**
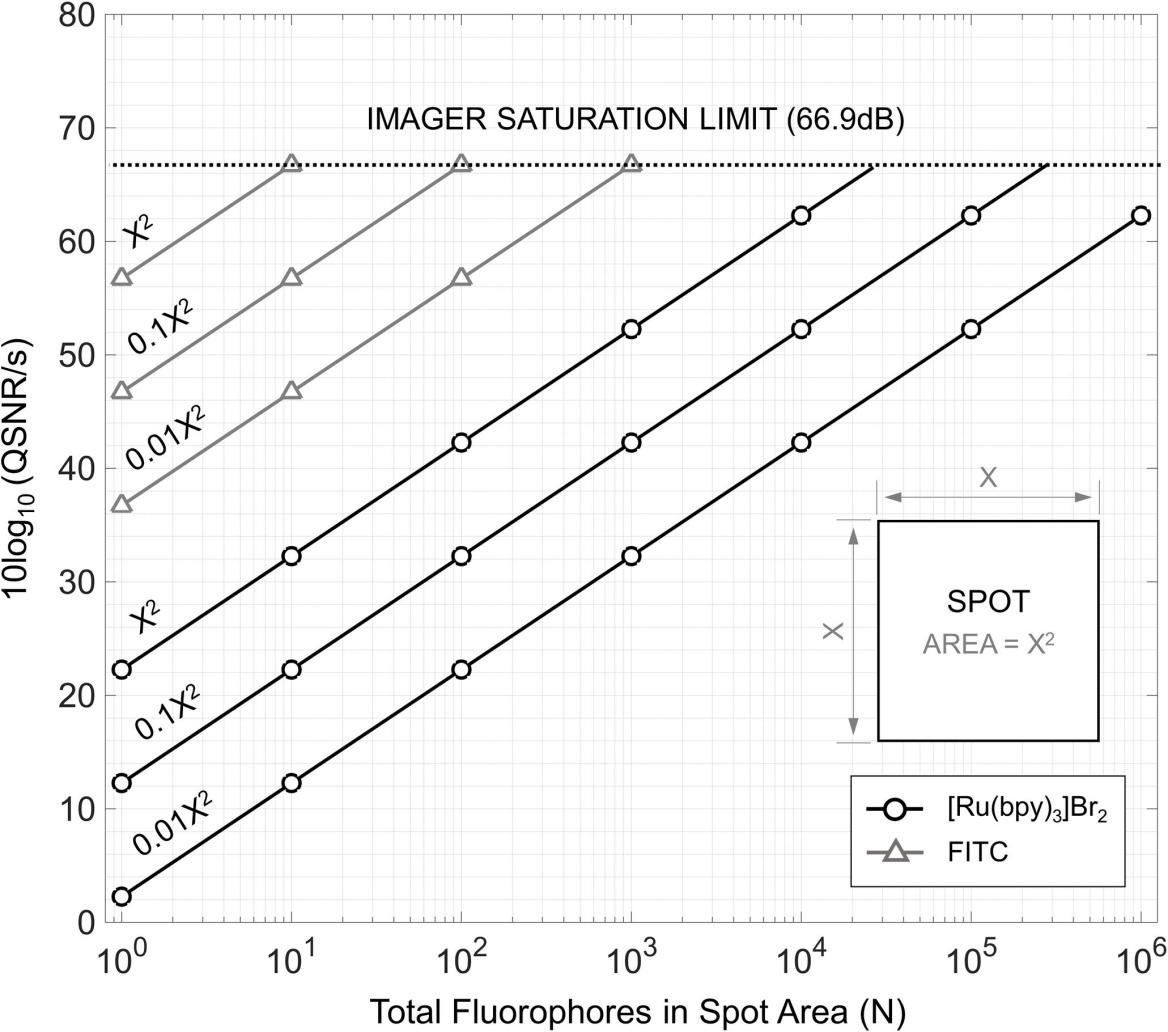
The quantum-limited SNR, normalized a total image acquisition time of 1s, is plotted as a function of spot scaling versus the number of fluorophores contained with the spot area for [Ru(bpy)_3_]Br_2_ and FITC fluorophores.

In order to study the SNR tradeoffs, we first analyze the quantum-limited SNR (QSNR) which is defined as the SNR in the absence of any extrinsic transduction or background noise, normalized to the total array image acquisition time, *T*_*C*_ [40]. The QNSR establishes the upper bound on the achievable SNR under theoretically perfect operating conditions. **Fig 10** plots the QSNR for this CW system for FITC and [Ru(bpy)_3_]Br_2_ fluorophores. The corresponding simulation conditions are outlined in **Table 4**. Additionally, we provide an analytical expression for the QSNR in **Table 5**. The achievable QNSR within one second of acquisition time is computed for different isomorphic spot scaling ratios, α. It can be observed that the QSNR decreases with decreasing α. As one attempts to image more spots on a given surface, the time that the imager can spend per spot decreases with *T*_*c*_α/2. Unsurprisingly, less photons are captured per spot thus negatively impacting the QSNR. To overcome this limitation, one must either increase the number of fluorophores within each spot, *N*, or *T*_*C*_.

**Table 5:** Closed-form formulations for signal-to-noise ratios.

Specification	Closed-Form Approximation
Quantum-limited SNR	$10\;\log_{10}\left(\frac{{G(\langle n_{p}\rangle \gamma_{e}\eta_{e})}^{2}}{{\sigma_{n}}_{p}^{2}\gamma_{e}\eta_{e}}\right)$
Continuous-wave SNR with imager noise and non-ideal optics	$QSNR-10\;\log_{10}\left(\frac{1}{1+\frac{\frac{1}{OD}\langle F_{x}\rangle T_{INT}\gamma_{x}\eta_{x}+\sigma_{r}^{2}+\beta T_{INT}}{\sigma_{n_{p}}^{2}\gamma_{e}\eta_{e}}}\right)$
Time-gated SNR with imager noise and non-ideal optics	$QSNR-10\;\log_{10}\left(\frac{1}{1+\frac{\frac{K\tau_{s}}{OD}\langle F_{x}\rangle {e^{-\frac{\Delta T_{D}}{\tau_{s}}}(1-e^{-\frac{T_{int}}{\tau_{s}}})\gamma }_{x}\eta_{x}+\sigma_{r}^{2}+\beta T_{INT}}{\sigma_{n_{p}}^{2}\gamma_{e}\eta_{e}}}\right)$

*〈n_*p*_〉 and $\sigma_{n_{p}}^{2}$ represent the equations for mean and variance of photons collected under CW or TG which are provided in **Section IV.** The imager emission coupling efficiency, γ, and quantum efficiency, η, are denoted with subscripts denote dependency on the emission (*e*) and excitation (*x*) wavelengths.

It is desirable to operate at the QSNR limit to achieve high dynamic range (DR) which directly trades off with the imaging acquisition time as observed in **Fig 10**. In practical system implementations the achievable SNR and DR can be significantly constrained due to extrinsic noise and signal transduction inefficiencies [37, 40]. For example, one primary noise source emanates from the imager’s transduction noise which is a combination of the electronic read noise and dark current leakage. Another prominent noise source arises from the presence of the excitation source which introduces background noise as imagers cannot distinguish excitation-emission wavelength differences with high selectivity. To combat background interference, precious imaging optics with high optical densities (OD) are commonly employed to reject indecent photons outside of the targeted fluorophore emission wavelength band [41, 42]. These optical components are often bulky, expensive, and complex and thus it is crucial that the right filter OD is selected for the application at hand. Finally, as the complexity of the optical components (e.g., coupling guides, focusing lenses) increase, the amount of photons coupled to the imaging pixels decreases substantially and can often be < 25% at state-of-the-art.

**Fig 11:**
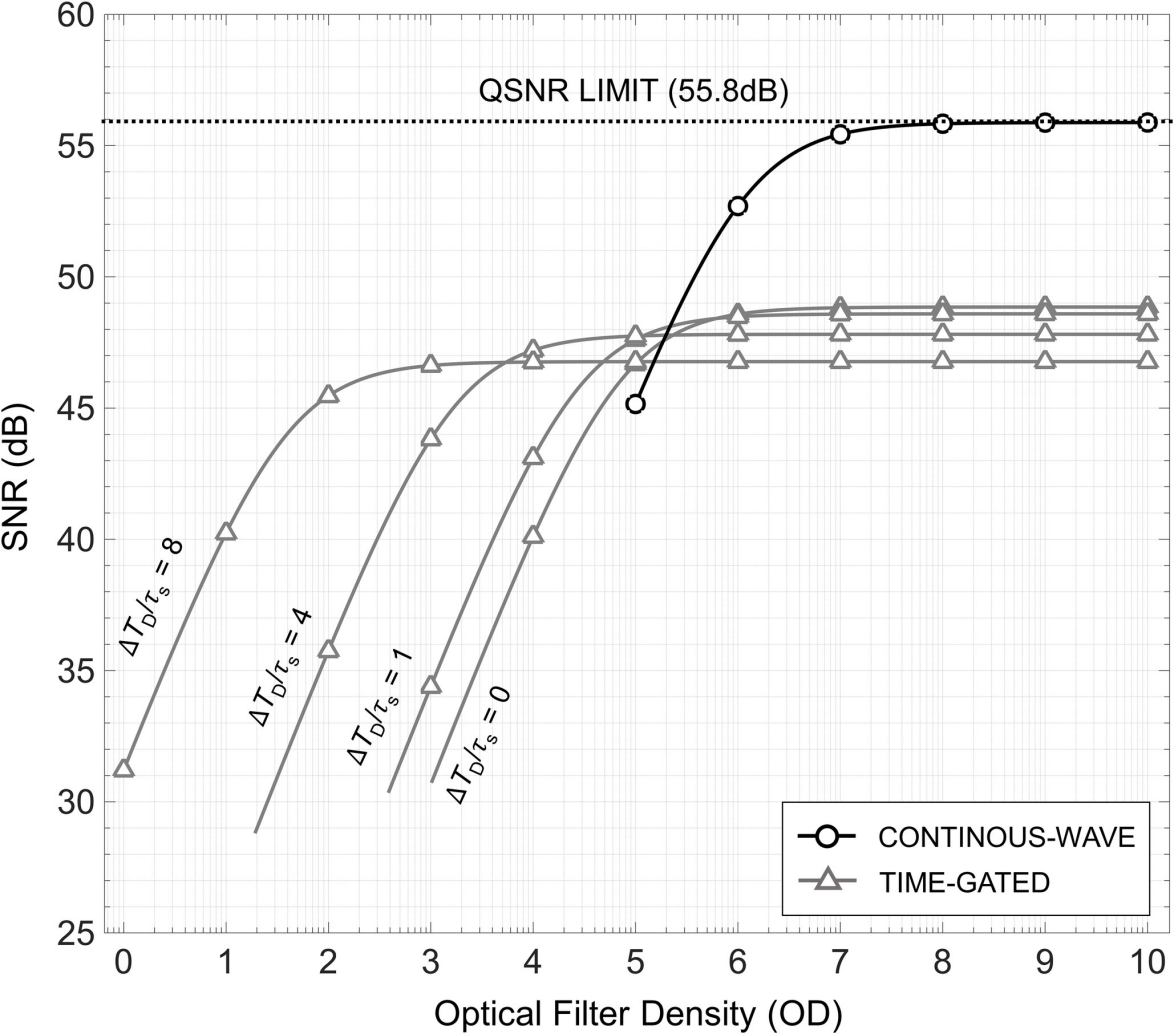
The impact of extrinsic noise sources on the measured SNR is plotted for both CW and TG against varying optical filter magnitudes. For TG, four different scenarios are plotted where the ratio between ΔT_*D*_ and *τ*_*s*_ is increased to observe enhanced background interference rejection.

To demonstrate the impact of optical components, specifically filter OD, and extrinsic noise sources on the SNR (see formulation in **Table 5**), **Fig 11** depicts the CW SNR for a fixed population of [Ru(bpy)_3_]Br_2_ fluorophores subject to different filter OD magnitudes. The simulation specifications are listed in **Table 7,** and the same imaging performance parameters listed in **Table 6** are used. The SNR curve exhibits two distinct regions. Initially, for low filter OD values, the SNR decays by 10 dB per decade with decreasing filter OD as the influence of the background noise competes with the measured *n*_*ph*_. As the filter OD is increased to higher values (> OD 6), the SNR begins to flatten and indicates that the background noise contribution is negligible, i.e., background-free. If the imaging hardware contributes negligible noise, adequate signal averaging is employed, and a large enough integration time is achieved, CW systems can produce measured SNRs that approach the QSNR limit.

**Table 6:** Imaging system performance specifications for example applications.

System Parameter	Value
Number of imaging pixels (G)	100
Imager emission coupling efficiency (γ)	1%
Imager quantum efficiency (η)	0.45
Imager pixel well-capacity (W)	50,000 e^-^
Input-referred electronic readout noise (*σ*_*r*_)	2 e^-^
Dark current leakage (β)	5 e^-^/s
Excitation decay time constant (*τ*_*S*_)	50 ns

On the other hand, it is common to employ TG over CW as it brings different advantages to the design space when considering proper selection of filter OD. **Fig 11** also depicts the SNR for a TG system with different Δ*T*_*D*_ (grey lines) subject to the same filter ODs used in the CW system. It should be noted that the number of TG measurements, *K*, in these examples is constrained by *T*_*C*_. At first glance, the magnitude of the background free SNR of a TG system is almost always lower than that of the CW system primarily because TG results in lower *n*_*ph*_. Additionally, because of the smaller *n*_*ph*_, one may have to accumulate more images than expected to acquire a discernable signal level otherwise the limit of detection could be compromised. Moreover, the complexity of a TG system can surpass that of CW as the lifetime of the fluorophore decreases since it requires high-bandwidth processing equipment. Despite these setbacks, a major advantage TG offers is that it extends the background free region over substantially lower filter OD (< OD 4) values thus increasing its DR in comparison to CW. This can be advantageous when attempting to physically scale down the size of an imaging system or operating with low-well capacity imagers.

### B. Excitation power requirements with non-ideal optics

**Fig 12:**
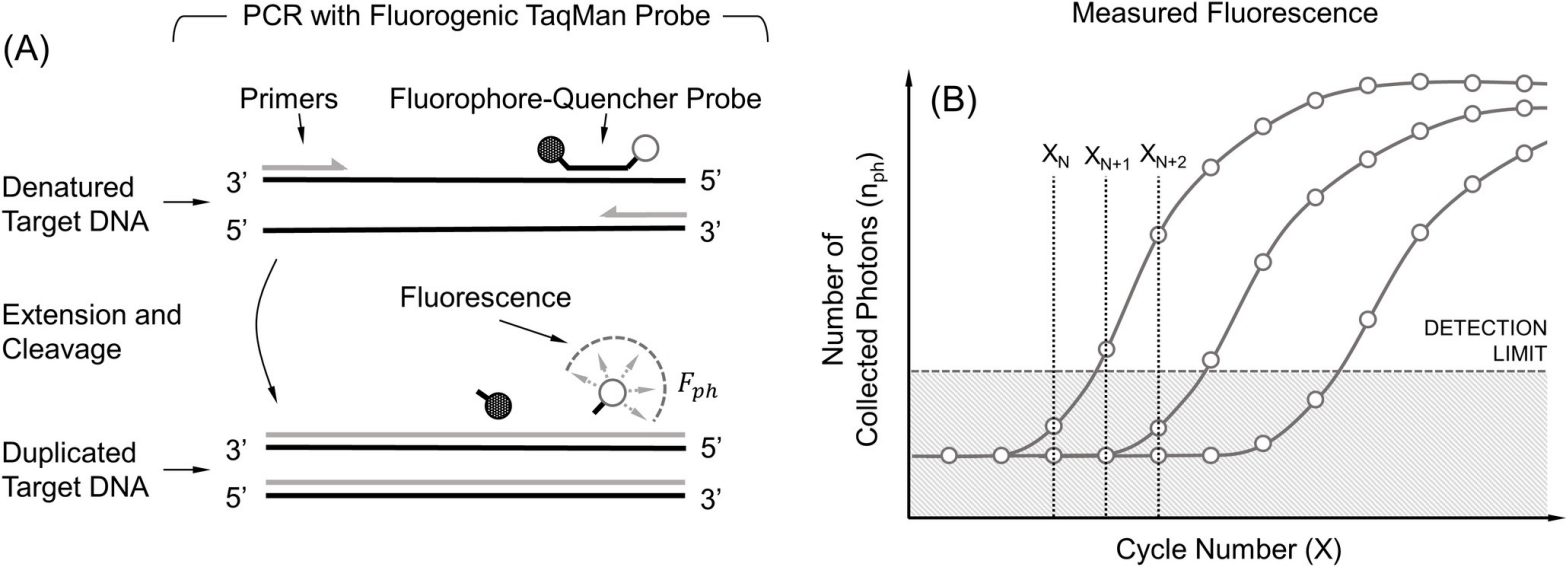
(A) Pictorial representation of RT-qPCR process with the fluorogenic TaqMan probe where fluorescence is generated upon successful extension of primers and cleavage of probe complex. (B) Example of typical RT-qPCR fluorescence measurements as a function of the amplification cycle number and concentration of fluorophores present.

While large-scale imaging systems may benefit from high precision optics, expensive scientific cameras, and relatively unlimited power, there are many instances where this is not always the case. For instance, the deployment of point-of-care (POC), implantable, or single-use systems tend to have stringent limits on cost, space, and available power supplies. Consequently, these systems encounter distinct tradeoffs between the achievable minimum number of detectable fluorophores, referred to as the minimum detection limit (MDL) [43, 44], and the required input excitation power when employing CW or TG. One practical example of a resource constrained system is POC real-time quantitative polymerase chain reaction (RT-qPCR) which is commonly employed for detection of viruses and/or bacteria [45]. **Fig 12** depicts how the fluorescence is generated using a fluorogenic probe [46] (TaqMan probe).

**Fig 13:**
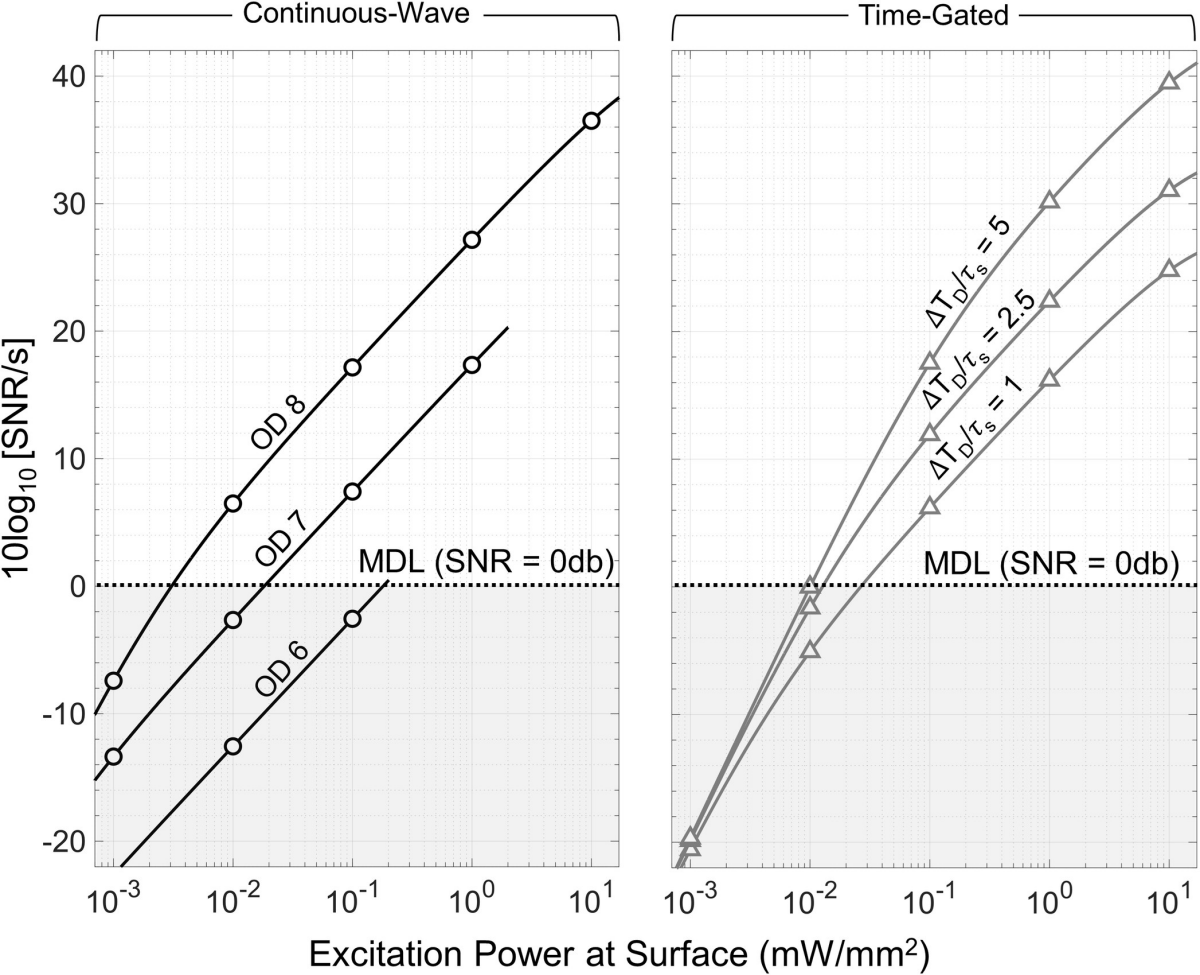
Predicted SNR normalized to the acquisition time of 1s versus input excitation power for CW and TG systems. In the CW case, three scenarios are considered where the utilized optical filter has an OD > 6. Conversely, in the TG case, the utilized optical filter is fixed at OD 4 while the ratio between ΔT_*D*_ and *τ*_*s*_ is swept.

**Fig 13** presents the SNR, normalized to the measurement time, *T*_*c*_, versus average input excitation power for both CW and TG where we define the MDL to be when the SNR = 0. Again, the number of TG measurements, *K*, in these examples is constrained by *T*_*C*_. The simulation is performed for a fixed population of Eu^3+^L_2_ fluorophores with the same general simulation specifications outlined in **Table 7**, however the decay time time-constant of the excitation source is increased to 500 ns to represent a leaky source as one might encounter with an LED or other non-laser source. The SNR for the CW system is plotted for filter OD 6–8 whereas the SNR for the TG system is plotted with a fixed OD 4 but with varying Δ*T*_*D*_. Over the range of excitation power, CW more readily achieves the specified MDL level with lower excitation power granted that it has a sufficiently high filter OD (> OD 8). In reality, many optical filters can suffer degradation to their OD due to material imperfections or environmental influences like high optical scattering. Therefore, it is advisable to consider CW performance under less-than-ideal OD conditions (< OD 6) which ultimately results in having to burn more excitation power to meet the MDL. However, a TG system, even with an OD 4, can meet with the specified MDL level with significantly less excitation power than that of a CW system with an OD 6 filter. In fact, with an appropriate Δ*T*_*D*_ and *T*_*int*_, the TG system can outperform a CW system equipped with an OD 8 filter at moderate to high excitation power levels.

**Table 7:** Simulation specifications for figure.

Simulation Parameter	Value
Spot diameter (d)	100 μm
Total image acquisition time (T_*C*_)	250 ms
Number of fluorophores in spot (N)	10,000
Number of fluorophores in excited state ($\bar{n_{e}}$)	100 (1%)
TG excitation pulse duration (T_*p*_)	4.2 μs (5*τ*_*l*_)
TG time delay (ΔT_*D*_)	0, 50, 200, 400 ns
TG total time window (Δ*T*_*D*_+*T*_*int*_)	5 μs (6*τ*_*l*_)

### C. Stochastic modulation through molecular interactions

**Fig 14:**
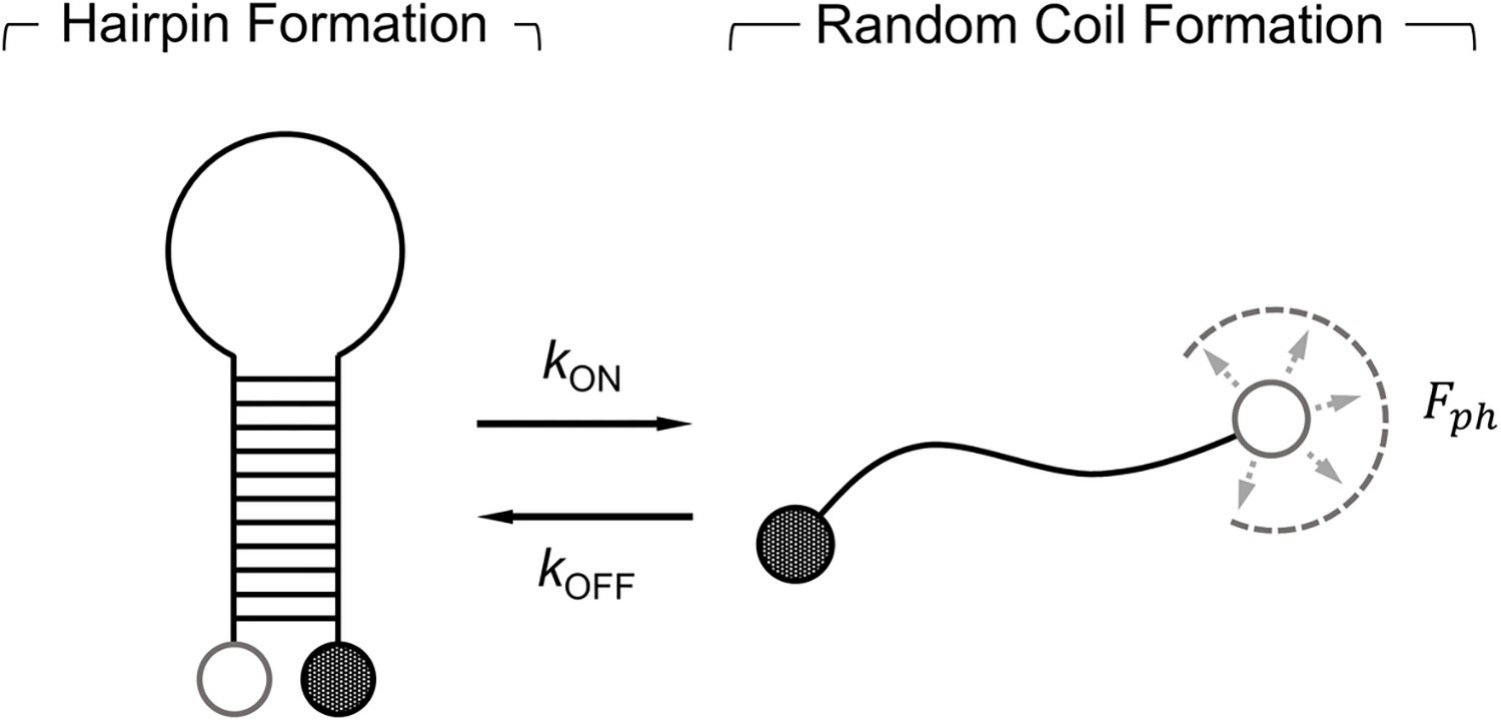
Depiction of the two possible quenched (hairpin) and unquenched (random coil) conformational states ssDNA can switch between at equilibrium.

In many applications, such as DNA hybridization assays [47], the observed fluorescence is modulated by external molecular interactions such as binding. Therefore, the spectral characteristics of the fluctuations in *F*_*ph*_ will be impacted. To study how the PSD derived in **Section IV.B** changes as a function of molecular interaction kinetics, let’s consider a simple system of a single-strand DNA (ssDNA) tagged with a fluorophore-quencher pair. As depicted in **Fig 14,** a ssDNA fragment will constantly change conformation between its unquenched (random coil) and quenched (hairpin) states. To express the influence of hairpin formation on the PSD of *F*_*ph*_, we first need to formulate the PSD describing the fluctuation of ssDNA fragments in their unquenched state.

Let’s consider a system at equilibrium where the concentration of ssDNA strands, [X_0_], is equal to the sum of the concentration of unquenched strands, [X_S1_], and quenched states, [X_S2_]. The rate of change in [X_S1_] can be expressed by the rates at which an strands forms or loses it hairpin structure denoted as k_on_ and k_off_, respectively

$$\left[X_{S1}]\underset{k_{on},k_{off}}{↔}[X_{S2}\right]$$ (32)

$$\frac{d[X_{S1}]}{dt}=k_{off}(\left[X_{0}]-[X_{S1}\right])-k_{on}[X_{S1}]$$ (33)

Therefore, the equilibrium concentration of unquenched strands, $\bar{\left[X_{S1}\right]}$, can be expressed in terms of [X_0_]

$$\bar{\left[X_{S1}\right]}=\frac{k_{off}}{k_{on}+k_{off}}[X_{0}]$$ (34)

With (33) and (34), it can be shown that the impulse response, *h*(*t*), of [*X*_*S*1_] when subjected to a small change in [*X*_*S*1_] is given by

$$h(t)=e^{-t(k_{on}+k_{off})}$$ (35)

Consequently, we can apply Carson’s Theorem [48] to formulate the PSD of [*X*_*S*1_] as

$$B_{x}(\omega )=4\pi {\left(\frac{k_{on}k_{off}}{k_{on}+k_{off}}\right)}^{2}\delta (\omega )+(\frac{1}{k_{on}+k_{off}})\frac{4(\frac{k_{on}k_{off}}{k_{on}+k_{off}})}{1+{\left(\frac{\omega }{k_{on}+k_{off}}\right)}^{2}}$$ (36)

Now, because quenching due to hairpin formation can be treated as a linear process, the resulting PSD of the modulated *F*_*ph*_ is given by the multiplication of (36) with the PSD of *F*_*ph*_ dervied in (26). Given there are *N* ssDNA strands in the solution the modulated PSD is given

$$M_{x}(\omega )=NS_{x}(\omega )B_{x}(\omega )$$ (37)

**Fig 15:**
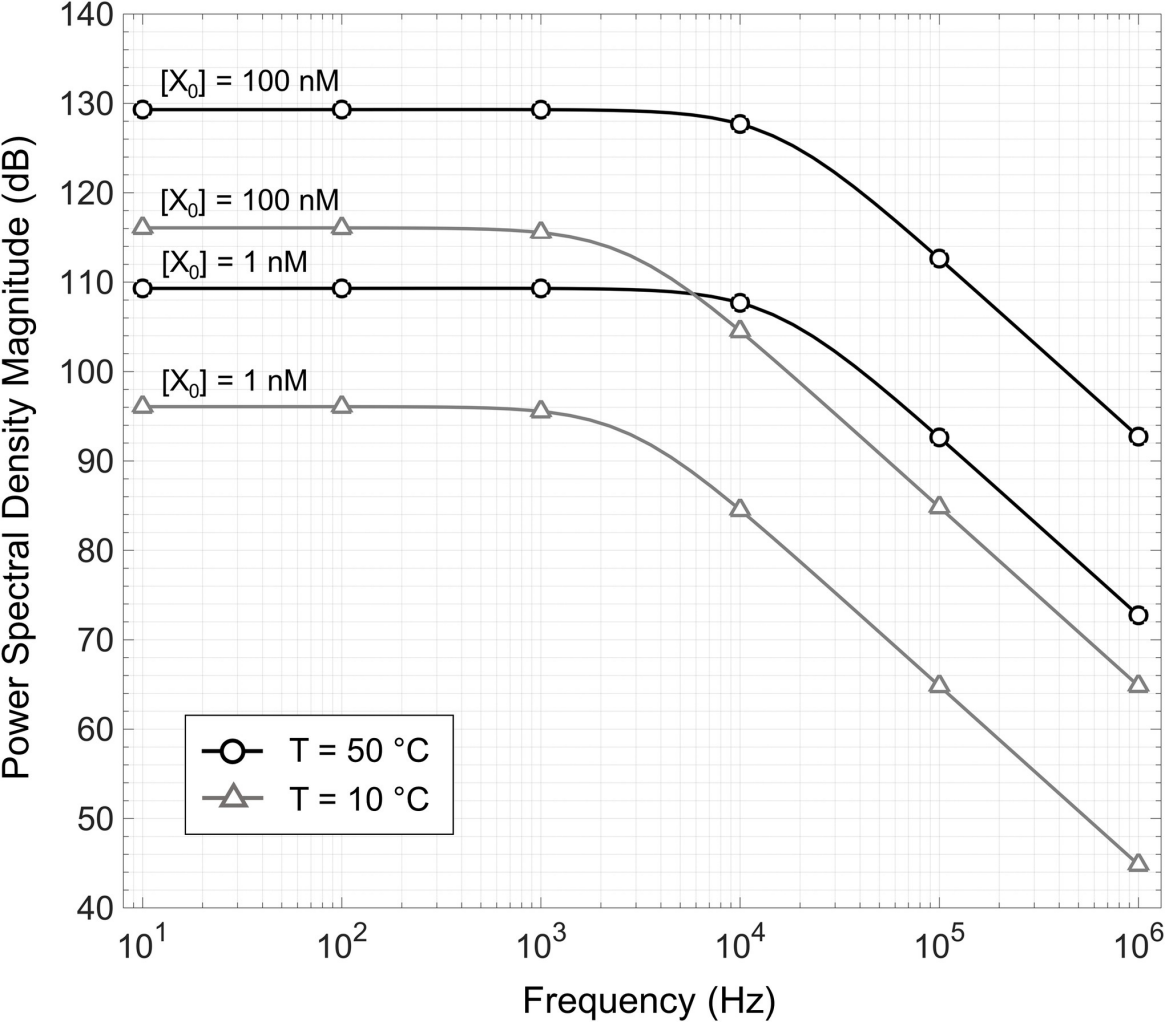
Predicted power spectral density of fluorescence under the influence of molecular binding kinetics for system with 1 nM and 100 nM of ssDNA against temperature.

**Fig 15** shows a simulation of (37) for varying concentrations of ssDNA with different temperatures. The simulation specifications and reaction kinetics are outlined in **Table 8**. The resulting PSD profile follows a Lorentzian profile with a spectral density proportional to the equilibrium value of unquenched ssDNA fragments and a 3dB corner frequency determined by (*k*_*on*_+*k*_*off*_)/2*π*. Moreover, the presence of binding imposes a bandwidth limit to the spectral characteristics of the PSD. This bandwidth influences the observed noise and appropriate integration time and thus should be considered when predicting the system performance metrics mentioned in the prior two examples.

**Table 8:** Simulation parameters of hairpin formation kinetics.

Simulation Parameter	Value(s)
Reaction Volume	25 μL
Concentration of ssDNA ([X_0_])	1 nM; 100 nM
ssDNA length	22 nt
Forward binding rate (k_on_)	5.5×10^4^ s^−1^(T = 50°C) 1.7×10^4^ s^−1^(T = 10°C)
Reverse binding rate (k_off_)	3.8×10^4^ s^−1^(T = 50°C) 2×10^2^ s^−1^(T = 10°C)

*Values extracted from [49]

It is imperative to recognize that the resulting PSD in (37) relies on the assumption of linearity of the quenching process. In most molecular beacon systems, such as the depicted ssDNA hairpin example, the dominant quenching process can be explained by static quenching which is exactly described by the complex rate formation in (32) [50]. However, in some systems, the quenching can be a combination of both static and dynamic quenching processes. We assume dynamic to be negligible here in this example; yet the presence of dynamic can introduce inverse quenching dependence as temperature and concentration are increased [14]. Thus, the overall quenching relationship might deviate from the linear case and lead to a more complex PSD than that presented in (37).

## VI. Conclusion

Fluorescence systems can be broken down into their ensemble average and stochastic components which are critical in determining the performance of optical biosensor systems. We present a stochastic modeling framework using ODEs and homogenous continuous-time Markov Chains, characterized by measurable photophysical properties of a fluorophore, and applied it to CW and TG measurements. We then distilled closed-form formulations to compute various expected performance metrics without the need for direct numerical simulation. Additionally, we employed the Markov chain to predict the fluctuations in emission photon flux under CW and TG measurements. Notably, we proved that fluctuations in the emission photon flux do not follow a Poisson random process and derived their spectral characteristics. Finally, we computationally verified the validity of the proposed modeling framework through Monte Carlo molecular dynamic simulations of a fluorescence system with the use of the Gillespie Algorithm.

The models presented throughout this work were used to identify system performance metrics such as quantum and extrinsically limited signal-to-noise ratios as well as detection limits for a given system. Furthermore, we elucidated core design tradeoffs involved in three different biosensing applications where we explored the influence of system scaling, imperfect optics, power constraints, and molecular interactions. In general, we observed that CW systems are superior in their limits of detection (i.e., high SNR), imaging speed, excitation power requirements, and ability to approach quantum-limited performance given adequate imaging hardware performance. However, these benefits all come at the price of a high OD filter which may not be feasible either due to cost/space constraints or from unavoidable complications imposed by material imperfections or environmental conditions. On the other hand, TG systems can substantially relax the filter OD requirement while still maintaining comparable SNR, acquisition speed, and required excitation power. In some cases, TG even outperforms CW systems outfitted with high OD optics indicating that TG systems should be carefully considered even if one has access to the correct optics. A critical consideration relates to the minimum number of TG measurements required to digitize a discernable reading which may not be possible depending on the fluorophore’s relaxation lifetime and the total time allotted to make the measurements. While it is crucial to select the proper measurement modality for the application at hand, it is even more important to judiciously select a fluorophore with photochemical qualities that complements the specified system metrics such as total acquisition time, power consumption, and or SNR.

While the system performance metrics did not consider molecular interactions, we demonstrated that these interactions do impact the observed fluctuation in the emission flux. Notably, we show that molecular binding band-limits the spectral characteristics of the emission flux which should be equally considered when predicting system performance or setting rational performance metrics.

Together, these insights allow one to better understand how system performance metrics can be prioritized and set within a variety of different biosensing applications. More importantly, the proposed modeling framework fuses the phenomena of fluorescence with the conventional design space by quantitatively identifying how certain photophysical characteristics impact the observed signal behavior. As one collects more information of the system’s behavior in a variety of environments, the model can progressively improve its predictive power and result in a deeper understanding of the fundamental limitations governing fluorescence detection.
